# In situ structural mechanism of epothilone-B-induced CNS axon regeneration

**DOI:** 10.1038/s41586-025-09654-z

**Published:** 2025-11-12

**Authors:** Satish Bodakuntla, Kenichiro Taira, Yurika Yamada, Pelayo Alvarez-Brecht, A. King Cada, Nirakar Basnet, Rui Zhang, Antonio Martinez-Sanchez, Christian Biertümpfel, Naoko Mizuno

**Affiliations:** 1 Laboratory of Structural Cell Biology, National Heart, Lung and Blood Institute, National Institutes of Health, Bethesda, MD USA; 2 Department of Computer Sciences, Faculty of Sciences, University of Oviedo, Oviedo, Spain; 3 Department of Biochemistry and Molecular Biophysics, School of Medicine, Washington University in St Louis, St Louis, MO USA; 4 Department of Information and Communications Engineering, Faculty of Computer Science, University of Murcia, Murcia, Spain; 5 National Institute of Arthritis and Musculoskeletal and Skin Diseases, National Institutes of Health, Bethesda, MD USA

**Keywords:** Cryoelectron tomography, Microtubules

## Abstract

Axons in the adult central nervous system (CNS) do not regenerate following injury, in contrast to neurons in the peripheral nervous system and neuronal growth during embryonic development. The molecular mechanisms that prevent regeneration of neurons in the CNS remain largely unknown^1,2^. Here, to address the intracellular response to injury, we developed an in situ cryo-electron tomography and cryo-electron microscopy platform to mimic axonal damage and present the structural mechanism underlying thalamic axon regeneration induced by the drug epothilone B. We observed that stabilized microtubules extend beyond the injury site, generating membrane tension and driving membrane expansion. Cryo-electron microscopy reveals the in situ structure of microtubules at 3.19 Å resolution, which engage epothilone B within the microtubule lattice at the regenerating front. During repair, tubulin clusters are delivered and incorporated into polymerizing microtubules at the regenerating site. These microtubule shoots serve as scaffolds for various types of vesicles and endoplasmic reticulum, facilitating the supply of materials necessary for axon repair until membrane tension normalizes. We demonstrate the unexpected ability of neuronal cells to adjust to strain induced by epothilone B, which creates homeostatic imbalances and activates axons to regeneration mode.

## Abstract (web summary)

A combined cryo-electron tomography and cryo-electron microscopy pipeline was developed to inflict axonal damage and monitor the cellular response induced by epothilone B, revealing that microtubule polymerization at and beyond the lesion site promote axon regeneration.

## Main

Axons in the CNS are susceptible to failure in regeneration following injury^1–3^. Patients with brain injury often experience functional and cognitive deterioration^4^, resulting in unfavourable outcomes such as paralysis and mortality. The challenges associated with axon regeneration arise from a combination of two key factors: the inhibitory microenvironment of the CNS and the intrinsic suppression in the process of axonal repair and regrowth. In particular, little is known about interconnected causalities at the cellular and molecular level; therefore, understanding the molecular determinants of axonal degeneration and the mechanisms that facilitate regeneration is critical. During axonal injury, damage to membranes allows the leakage of cellular components, triggering several key events to mitigate the damage^5^. Ca^2+^ influx occurs immediately^6–9^, leading to an ionic imbalance that triggers secondary reactions, such as mitochondrial dysfunction and microtubule depolymerization, presumably owing to accelerated catastrophe, a rapid phase of microtubule depolymerization. When the damage at the plasma membrane is repaired, further leakage is prevented. After injury, most axons exhibit bulb-like morphologies, termed retraction bulbs, at the tips of closed injury sites^10,11^, which are considered hallmarks of axons that will not regenerate.

Several studies have revealed a critical role for neuronal cytoskeleton dynamics in regeneration behaviours and outcomes. Cytoskeletal elements, including microtubules and actin, have been implicated in axon stability and neuroregeneration^12–17^. In particular, microtubule-stabilizing agents, such as Taxol or epothilone B (EpoB), have been examined for their effects on axon regeneration in the CNS^18–20^. Of particular interest is the use of EpoB as this US Food and Drug Administration (FDA)-approved anti-cancer compound is able to pass through the blood–brain barrier, unlike conventional Taxol compounds^21^. In vitro structural studies of Taxol and epothilone derivatives showed that they bind β-tubulin on the inner microtubule lumen^22–24^. Both Taxol and EpoB stabilize microtubule assembly by forcing tubulin dimers into a straight conformation, which is energetically favourable for microtubule formation and elongation. The administration of pharmacologically relevant quantities of EpoB induces axonal repair in adult mice following spinal cord injury^20^; however, the molecular basis of EpoB-induced axonal regeneration remains unknown.

In this study, we developed a platform that mimics axon injury (axotomy) on electron microscopy grids, facilitating direct observation of intracellular reorganization in injured axons. We revealed that regeneration is facilitated by hyper-polymerizing microtubule shoots, which maintain axonal microtubule identity and are stabilized by EpoB, as observed at near atomic resolution using cryo-electron microscopy (cryo-EM). These shoots aid in trafficking components for membrane repair, acting as rails until actin growth cones reform and membrane tension normalizes. This platform offers insights into axon repair mechanisms and has application in testing therapeutic agents, revealing unexpected neuronal plasticity and altered molecular events for axon regeneration.

## Cryo-EM platform for axon injury model

**Fig. 1:**
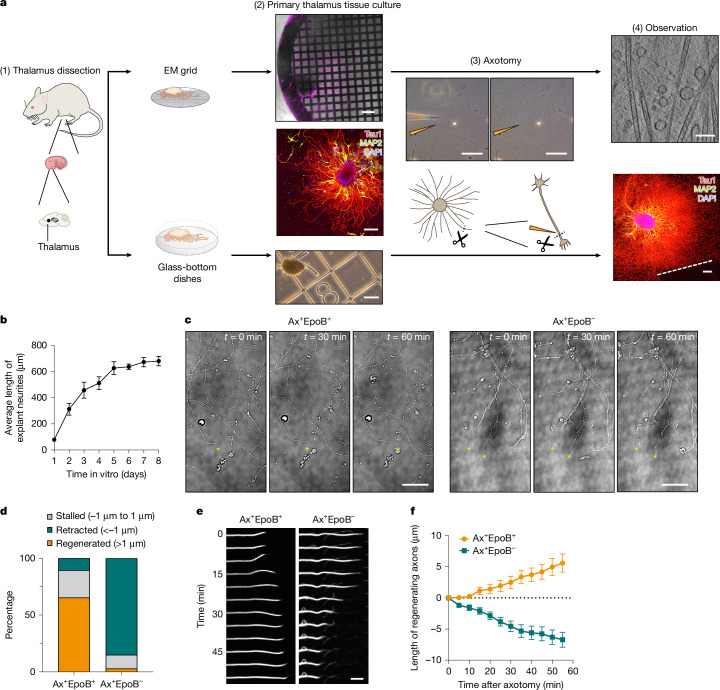
Integrative platform for axotomy and visualization of EpoB-mediated axon regeneration. **a**, The experimental pipeline developed in this study. (1) Thalamus tissue was dissected from embryonic day 15.5 (E15.5) embryos and cultured on electron microscopy (EM) grids (top) and glass-bottom dishes (bottom). (2) Explants extended axons onto EM grids (top; scale bar, 200 µm) or glass-bottom dishes (bottom; scale bar, 200 µm), showing axon-dominant neurite extensions (middle; scale bar, 100 µm). (3) Upon maturation, a thin needle was used to cut the targeted axon (scale bar, 50 µm); the overall procedure is shown in Supplementary Video 1. (4) Cryo-EM or cryo-ET (top; scale bar, 200 nm) and light microscopy (bottom; scale bar, 100 µm) analyses were performed. **b**, Explant growth on electron microscopy grids. Explant images were acquired at different time points using a 10× objective and the longest neurite length from the explant centre was measured. *n* = 20 (DIV 1), *n* = 16 (DIV 2), *n* = 15 (DIV 3), *n* = 14 (DIV 4), *n* = 15 (DIV 5), *n* = 26 (DIV 6), *n* = 14 (DIV 7) and *n* = 12 (DIV 8). Data are mean ± s.e.m. **c**, Axon regeneration in the presence of EpoB recorded by differential interference contrast microscopy, showing the neurite tip at axotomy (Ax, *t* = 0 min, asterisk). Scale bars, 20 µm. Video of regenerating axons (Ax^+^EpoB^+^) and retracting or stalled axons (Ax^+^EpoB^−^) (control) are available in Supplementary Video 2. **d**, Quantification of axon reactions after axotomy. *n* = 29 (Ax^+^EpoB^+^) and *n* = 33 (Ax^+^EpoB^−^) axons. **e**, Snapshots of regenerating axons labelled with SiR-tubulin (Ax^+^EpoB^+^ and Ax^+^EpoB^−^). The mild microtubule-stabilizing effect of SiR-tubulin was negligible in the control (Ax^+^EpoB^−^) axons. Scale bar, 5 µm. **f**, Quantification of axon length over time. Start point is the axotomy site. Data are mean ± s.e.m. *n* = 29 (Ax^+^EpoB^+^) and *n* = 33 (Ax^+^EpoB^−^). Source data

**Extended Data Fig. 1:**
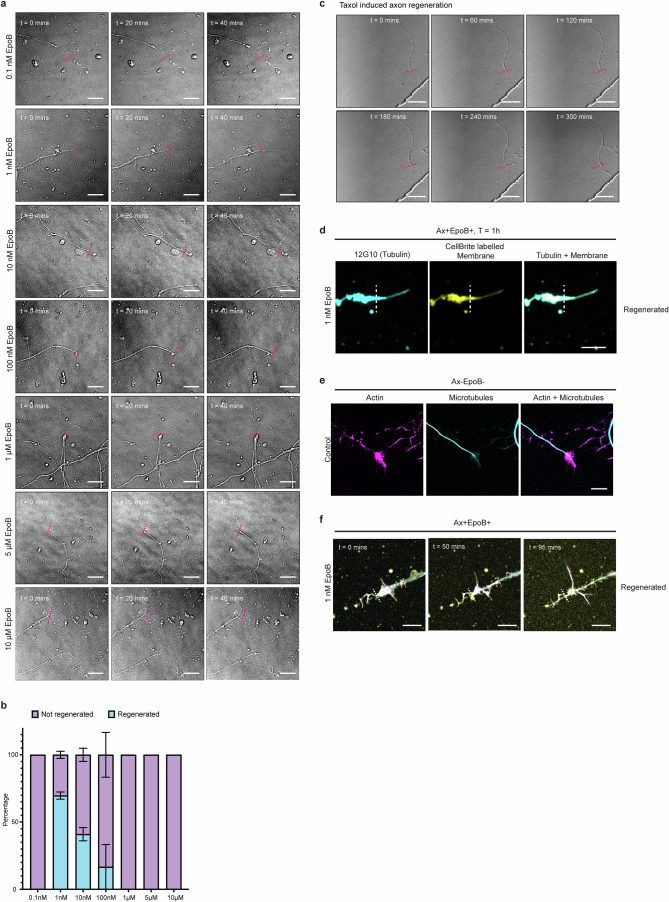
EpoB dose response and cytoskeletal organization in regenerating axons. a) Representative images of regenerating thalamus axons treated with different doses of EpoB shown at different time points. Magenta dotted lines indicate the cut site. Scale bar: 10 µm. Corresponding movies are shown in Supplementary Video 3. b) Quantification of regeneration in response to different doses of EpoB. The stacked bar graph shows mean as a horizontal line ± s.e.m. *n* = 7 (0.1 nM), *n* = 13 (1 nM), *n* = 14 (10 nM), *n* = 16 (100 nM), *n* = 17 (1 µM), *n* = 16 (5 µM) and *n* = 13 (10 µM) axons. c) Representative images of regenerating thalamus axons treated with taxol shown at different time points. Magenta dotted lines indicate the cut site. Scale bar 20 µm. The corresponding movie is shown in Supplementary Video 4. d) Immunofluorescence image of thalamus axons after axotomy and treated with EpoB for 1 h, fixed and stained with tubulin antibody 12G10 (cyan) and CellBrite membrane labeling dye (yellow). The axons were not permeabilized to maintain membrane integrity. Scale bar: 5 µm. e) Control thalamus explants stained with SPY555-actin (magenta) and SPY650-tubulin (cyan). Scale bar: 5 µm. f) Regeneration test of thalamus explants after axotomy at different time points. The axons were stained with SPY650-tubulin (Cyan, microtubules), SPY555-actin (magenta, plasma membrane) and MemGlow 488 (plasma membrane, Yellow). Scale bar: 5 µm. The corresponding videos are shown in Supplementary Video 5. Source data

To gain insights into the molecular mechanisms that underlie axonal regeneration following injury, we established a two-pronged approach combining cryo-EM and cryo-electron tomography (cryo-ET) to visualize the ultrastructure within regenerating axons and light microscopy-based observations to measure specific growth dynamics (Fig. 1a). We dissected CNS thalamic tissue from the brain of 15.5-day-old mouse embryos (E15.5) and grew explants on specimen grids for cryo-EM or glass dishes for light microscopy (Fig. 1a). Axon growth was prominent during the initial stages of explant formation; the axons matured and the growth rate reached a plateau after 6–8 days in vitro (DIV) (Fig. 1b). After axon growth plateaued, we performed axotomy (Supplementary Video 1) by removing distal axons that were more than 250 µm away from the soma with a glass capillary using a micromanipulator. We then characterized axon regeneration with and without EpoB by a combination of light and electron microscopy (Fig. 1a,c–f and Supplementary Video 2). Under control conditions without EpoB, most injured axons degenerated or stalled (84% retracted, 12% stalled,* n* = 33), with no observed growth or regeneration within 1 h of axotomy. By contrast, when EpoB was administered immediately following axotomy, using the same concentration range as in a previous mouse study (approximately 1 nM) and corresponding to concentrations administrated to patients with breast cancer^20,25^, visible regeneration was observed following a latency period lasting approximately 10 min (66% regenerated, 24% stalled, *n* = 29; Fig. 1c–e and Supplementary Video 2). However, EpoB-induced axon regeneration was dose-dependent; doses of 1 nM and 10 nM promote regeneration, whereas lower (0.1 nM) and higher (100 nM–10 µM) doses had no effect or were detrimental to injured axons (Extended Data Fig. 1a,b and Supplementary Video 3). As a control, we validated the occurrence of axon regeneration by administering 1 nM of Taxol (Extended Data Fig. 1c and Supplementary Video 4).

## Membrane stretching at regenerating site

**Fig. 2:**
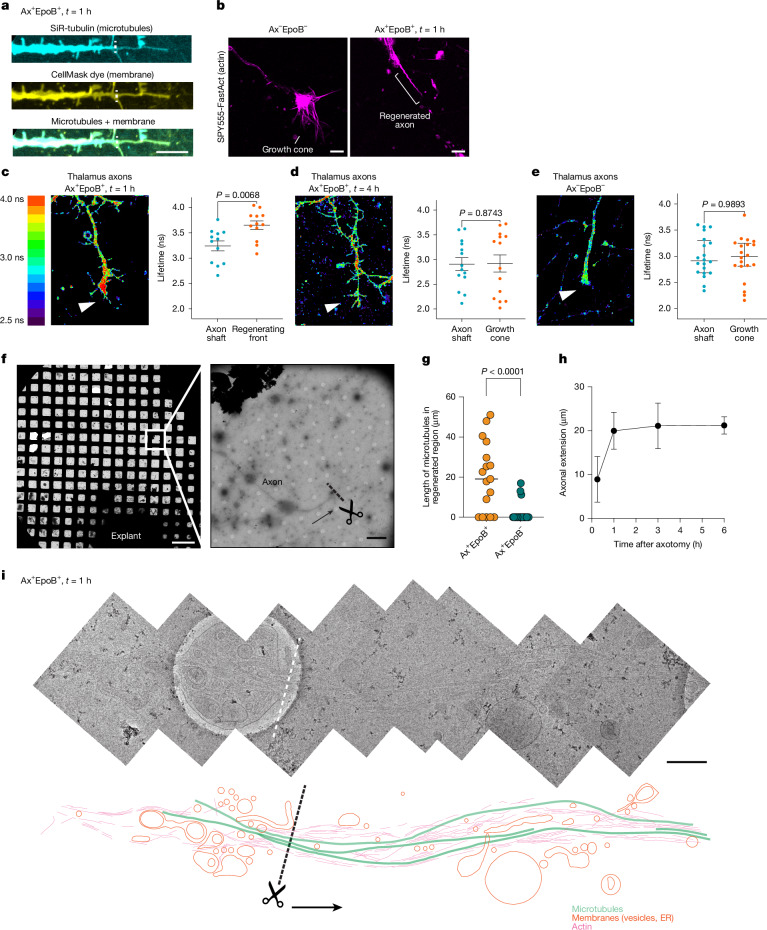
Cytoskeletal organization and membrane tension dynamics during EpoB-induced axon regeneration. **a**, Colocalization of microtubule (SiR-tubulin) and membrane (CellMask) signals at regenerating axon tips 1 h after axotomy and EpoB treatment. Scale bar, 10 µm. **b**, Actin organization (SPY555-FastAct) at the tip of axons under control (Ax^−^EpoB^−^) and regenerating (Ax^+^EpoB^+^) conditions. Scale bars, 3 µm. **c**–**e**, Membrane tension analysis by FLIM. **c**, Membrane tension is increased at tips relative to shafts (>15 µm downstream from the tip) 1 h after axotomy (*n* = 12). **d**, Tension normalizes at 4 h (*n* = 14). **e**, Control neurons show no difference between tip and shaft (*n* = 20). Data are mean ± s.e.m.; two-tailed Mann–Whitney test. **f**, Cryo-EM images of regenerating axons. The grid view gives an overview of the location (left; scale bar, 200 µm), with a magnified view showing an axotomy site (right; scale bar, 5 µm). Arrow indicates axonal growth direction and dashed line and scissors indicate the axotomy site. **g**, Distribution of microtubule lengths in cryo-EM snapshots 1 h after axotomy. Ax^+^EpoB^+^: *n* = 17, median = 19.1; Ax^+^EpoB^−^: *n* = 21, median = 0. The centre line shows the median; two-tailed Mann–Whitney test. **h**, Time course of axonal extension by cryo-EM snapshots, in the presence of EpoB after axotomy. *n* = 6 (15 min), *n* = 17 (1 h), *n* = 6 (3 h) and *n* = 12 (6 h) axons. Data are mean ± s.e.m. **i**, High-magnification montage of a regenerating axon 1 h after axotomy with EpoB. Top, cryo-EM montage; dashed line indicates the axotomy site. Scale bar, 400 nm. Bottom, segmentation depicting microtubules (green), membranes (vesicles and endoplasmic reticulum (ER)) (orange) and actin (magenta). Arrow depicts axonal growth direction. Source data

We visualized the tip of regenerating axons by live imaging of neurons (Fig. 2a), as well as by fixed staining (Extended Data Fig. 1d) 1 h post-axotomy in the presence of EpoB. Unlike control axons, which typically display an actin-rich growth cone, regenerating axons lacked this structure (Fig. 2b and Extended Data Fig. 1e). Instead, the tubulin signal extended directly up to the plasma membrane at the regenerating front (Fig. 2a, Extended Data Fig. 1d and Supplementary Video 5). This observation led us to hypothesize that the extended microtubules may mechanically stretch the membrane owing to the absence of actin at the growth cone. To test this, we used a mechanosensitive membrane dye, Flipper-TR^26^, which monitors membrane tension based on changes in fluorescence lifetime (a longer lifetime indicates higher membrane tension), using fluorescence lifetime imaging microscopy (FLIM). At 1 h after EpoB-induced regeneration, the fluorescence lifetime at the axon tip was significantly longer than at the axon shaft of the same axon (3.64 ns at the axon tip versus 3.24 ns at the axon shaft), indicating increased membrane tension at the regenerating site (Fig. 2c). At 4 h, the difference became insignificant (2.91 ns versus 2.90 ns) (Fig. 2d), showing that membrane tension had normalized. The membrane tension measured at the growth cone at 4 h was similar to that observed in normal growing axons (2.99 ns versus 2.91 ns) (Fig. 2e). Therefore, we concluded that membrane tension is transiently increased during early stages of regeneration due to the extension of microtubules towards the membrane surface, and gradually resolves as the regeneration progresses.

## Regeneration driven by microtubule shoots

**Extended Data Fig. 2:**
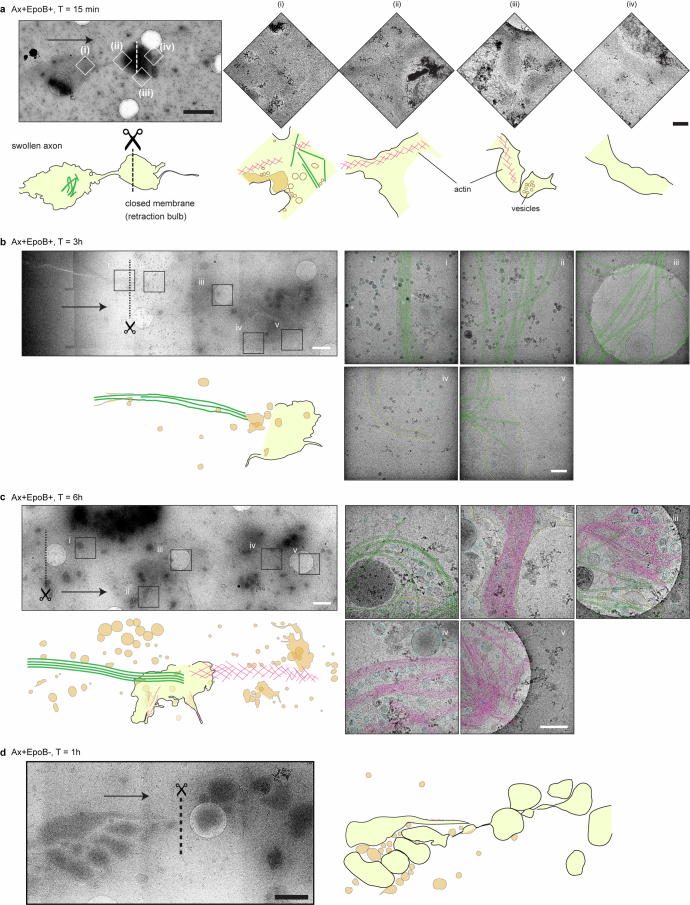
Time course of ultrastructural changes in regenerating axons with and without EpoB. a) Axon injury site in the presence of EpoB, 15 min after axotomy. Top left: Cryo-EM snapshot with a white dotted line indicating the axotomy site and white boxes indicating positions of magnified insets. The black arrow indicates the axonal growth direction. Scale bar: 2 µm. Top right: Magnified images of square insets. Scale bar: 200 nm. Bottom left: Segmentation of axon borders from image above, which appears swollen, showing a retraction bulb-like morphology. Bottom right: Segmentation of axon borders and visible cellular components from magnified insets above with microtubules (green), actin (magenta), membranes (orange) and vesicles (brown circles). b) A regenerating axon in the presence of EpoB 3 h after axotomy. Top: cryo-EM image of a regenerating axon beyond the axotomy site (black dotted line). The black arrow indicates the axonal growth direction. Scale bar 1 µm. Zoomed-in images of the boxes i-v are shown on the right. Scale bar: 100 nm. Bottom: Schematic depicting the border and visible cellular components at this magnification. Microtubules are shown in green and other membranous structures are in light yellow or orange. c) A regenerating axon in the presence of EpoB 6 h after axotomy. Top: cryo-EM image of a regenerating axon beyond axotomy site (black dotted line). The black arrow indicates axonal growth direction. Scale bar 1 µm. Zoomed-in images of the boxes i-v are shown on the right. Scale bar: 100 nm. Bottom: Schematic depicting the border and visible cellular components at this magnification. Microtubules are shown in green, actin in magenta and other membranous structures are in light yellow or orange. The tip of microtubule-shoots is topped by actin bundles. d) A tip of an axon in the absence of EpoB, 1 h after axotomy. Top: Low magnification cryo-EM image. The black arrow indicates axonal growth direction. Scale bar: 1 µm. Bottom: Schematic of visible structures. Actin is shown in magenta and membranous structures are in light yellow or orange.

**Extended Data Fig. 3:**
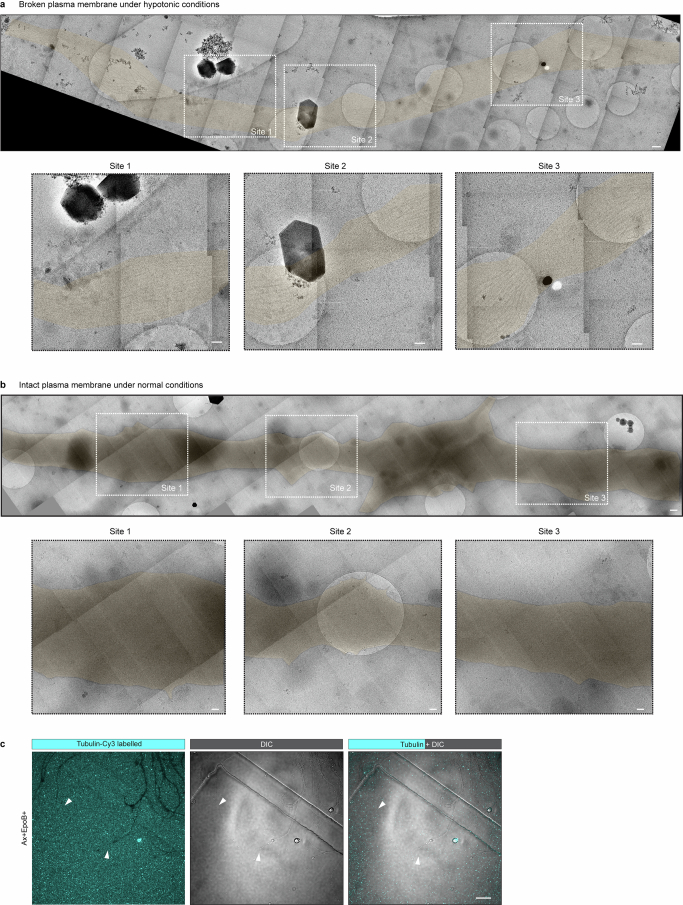
Quality control of cryo-EM images showing membrane rupture under high tension. a) Cryo-EM montage of a thalamus axon incubated in hypotonic media conditions before vitrification. The top panel shows a high-magnification montage of an axon. Under hypotonic conditions, membrane tension increases and compromises membrane integrity during the vitrification process. Scale bar: 400 nm. Dotted white lines indicate the positions of zoomed-in insets shown below the montage. A light orange shade highlights the trace of the axon. Scale bar: 200 nm. b) High-magnification cryo-EM montage of a control axon (neurons grown in normal culture media). Scale bar: 400 nm. Zoomed-in insets at different locations were shown below the montage. A light orange shade is used to highlight the trace of the axon. Scale bar: 200 nm. c) Live imaging of axons after axotomy and treated with EpoB. Fluorescently-labelled tubulin antibody (cyan) was added. No microtubule staining, showing the plasma membrane was sealed immediately after axotomy. Scale bar: 20 µm.

To gain a deeper understanding of the repair process at the axon tip, we vitrified regenerating axons at varying time points following axotomy: immediately (15 min) and after 1–24 h, and analysed the process using cryo-EM (Fig. 2f). Immediately after axotomy, injured axonal membranes were sealed (Extended Data Fig. 2a) and exhibited a retraction bulb morphology, despite the presence of EpoB (Extended Data Fig. 2). Ultrastructural observation by cryo-EM of the retraction bulb showed various cellular components, such as vesicles and actin (Extended Data Fig. 2a). Microtubules were not readily visible when examining the injured site towards the proximal direction, consistent with the general neuronal degeneration process in which microtubules depolymerize^13^. In the presence of EpoB at 1 h after axotomy, we observed bundles of microtubules shooting outward from the injury site (Fig. 2g–i and Supplementary Video 6). These microtubules extended 19.1 µm (median, Fig. 2g,h) beyond the damage site, similar to the typical growth rate of thalamic axons during the initial fast-growing phase^27^. These cryo-EM images of microtubule shoots extending from injury sites lacked discernable plasma membrane (Fig. 2i). This phenomenon was specific to regenerating sites, as control neurons without injury had an intact plasma membrane. The defect is likely due to the increased membrane tension in regenerating axons, leading to ruptured membranes during the cryo-preparation process. To verify this notion, we vitrified control neurons under hypotonic conditions, thus increasing membrane tension, which resulted in axons lacking plasma membrane (Extended Data Fig. 3a,b). In parallel, we verified membrane resealing after axotomy at the regenerating sites by co-labelling membranes and microtubules during regeneration (Fig. 2a) and using non-cell-permeable tubulin antibodies, resulting in an axon interior devoid of fluorescent signal as seen by confocal microscopy (Extended Data Fig. 3c).

## Shoots adopt axonal microtubule pattern

**Fig. 3:**
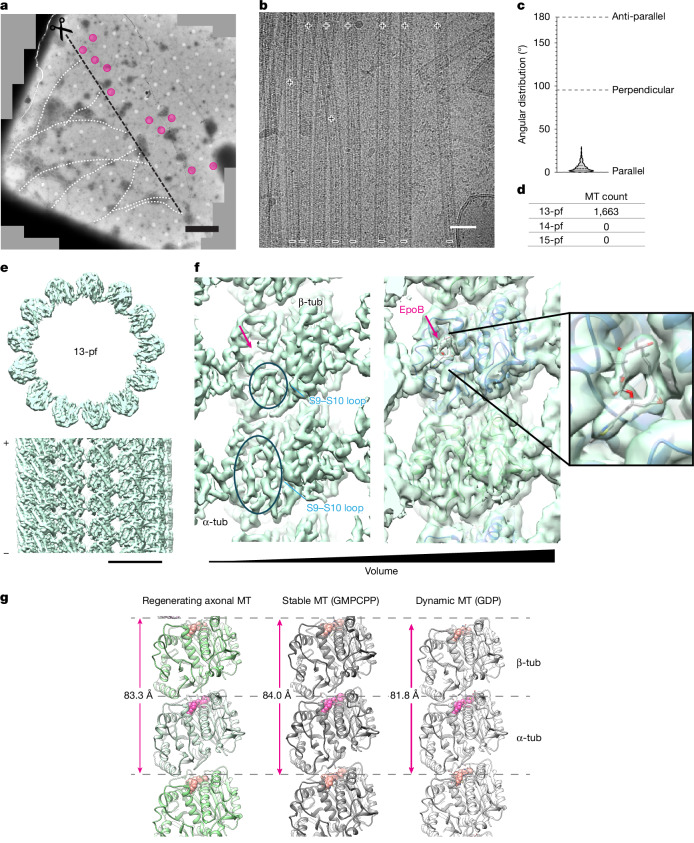
In situ cryo-EM reconstruction of microtubule shoots in regenerating axon. **a**, Large-scale axotomy of thalamus axons and regeneration in the presence of EpoB. Cryo-EM grid square 1 h after axotomy shows axons (white dotted lines) and the scission line (black dashed line). Beyond the cut, regenerating axons were visible. Magenta circles indicate grid holes with microtubule shoots used for SPA. Scale bar, 10 µm. **b**, Representative cryo-EM image of microtubule shoots taken for SPA. The polarities of the microtubules are indicated by + (plus end) and – (minus end). Scale bar, 50 nm. The corresponding tomogram is shown in Supplementary Video 6. **c**, Distributions of microtubule angles compared to reference microtubules. Angles close to 0° indicate polarities aligned in parallel, whereas angles close to 180° indicate an anti-parallel alignment. *n* = 1,663 microtubules, 4,123 segments. **d**, Distribution of microtubule protofilament (pf) numbers among observed microtubules (MTs). All 1,663 microtubules display the physiologically relevant 13-protofilament arrangement. **e**, 3D reconstruction of microtubule shoots at 3.19 Å resolution, showing the 13-protofilament arrangement. The reconstruction before focused refinement was used for display. Scale bar, 100 Å. **f**, 3D reconstruction of microtubule shoots, highlighting the inner lumen of microtubules. Left, B-factor sharpened map, with clearly distinguishable separation of α-tubulin (α-tub) and β-tubulin (β-tub) subunits within a dimer unit. Circles depict the S9–S10 loops and the magenta arrow indicates the EpoB binding pocket. Middle, higher volume threshold without sharpening, showing EpoB in the binding pocket. Right, magnified view of EpoB in the binding pocket. **g**, Left, atomic model of the microtubule shoots with an inter-dimer distance of 83.3 Å. For comparison, GMPCPP-stabilized microtubules adopt a stretched-dimer conformation (middle; inter-dimer distance 84.0 Å (Protein Data Bank (PDB): 6DPU)) and GDP-bound dynamic microtubules have a relaxed conformation (right; inter-dimer distance 81.8 Å (PDB: 6DPV)). EpoB-induced microtubule shoots adopt a stretched–stabilized lattice. Pink, α-tubulin nucleotide; salmon, β-tubulin nucleotide. Source data

**Extended Data Fig. 4:**
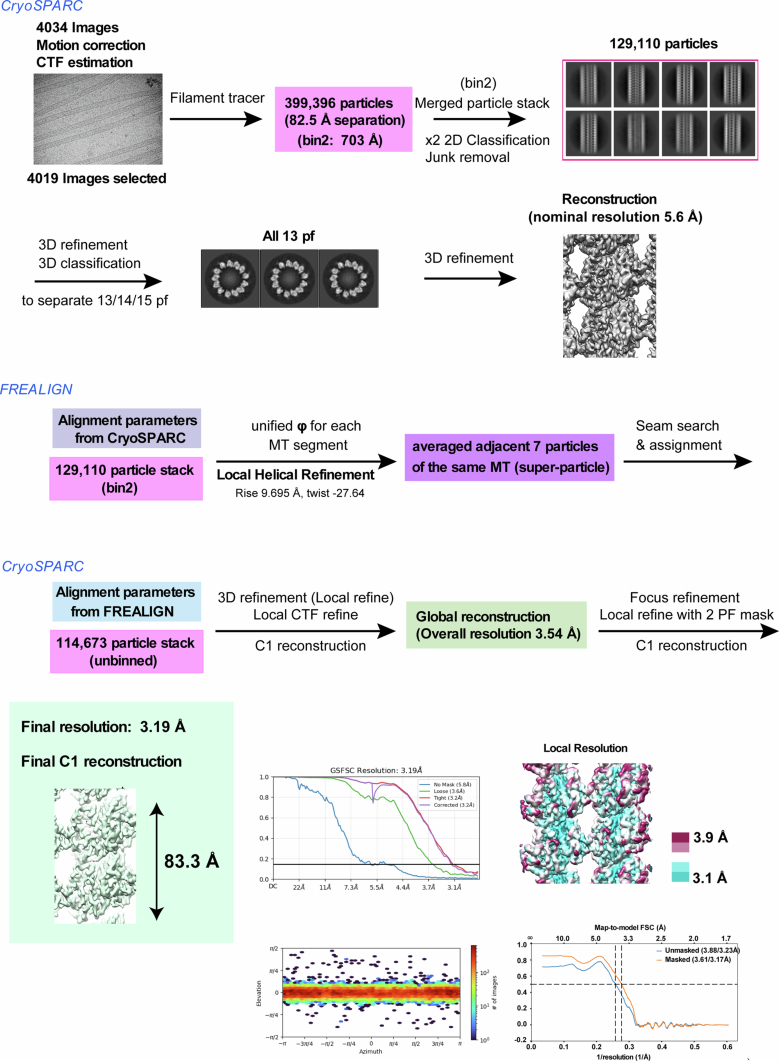
SPA processing workflow for high-resolution reconstruction of microtubule shoots 1 h after axotomy. The 3D reconstruction was performed using FREALIGN and CryoSPARC. Using CryoSPARC, microtubules were segmented into 399,396 overlapping boxes (703 Å) with an 82.5 Å step size, corresponding to the tubulin heterodimer length. After alignment and 2D classification in CryoSPARC, 129,110 particles with clear filament features were retained for 3D classification and 3D reconstruction. All analyzed microtubules (n = 1663) exhibited a 13-protofilament architecture. Seam detection was conducted in FREALIGN v9.11 using previously established protocols^73^. Final reconstructions were performed using 114,673 particles with the identification of clear seam position using CryoSPARC. The reconstruction was performed in C1 without imposing pseudo-helical symmetry, preserving the α/β-tubulin distinction. After per-particle defocus refinement and focused refinement, the analysis yielded a C1 reconstruction at 3.19 Å resolution (gold-standard FSC at 0.143) visualizing a clear EpoB engagement. GSFSC, Local resolution map, orientation distribution and map-to-model FSC are included in the figure.

**Extended Data Fig. 5:**
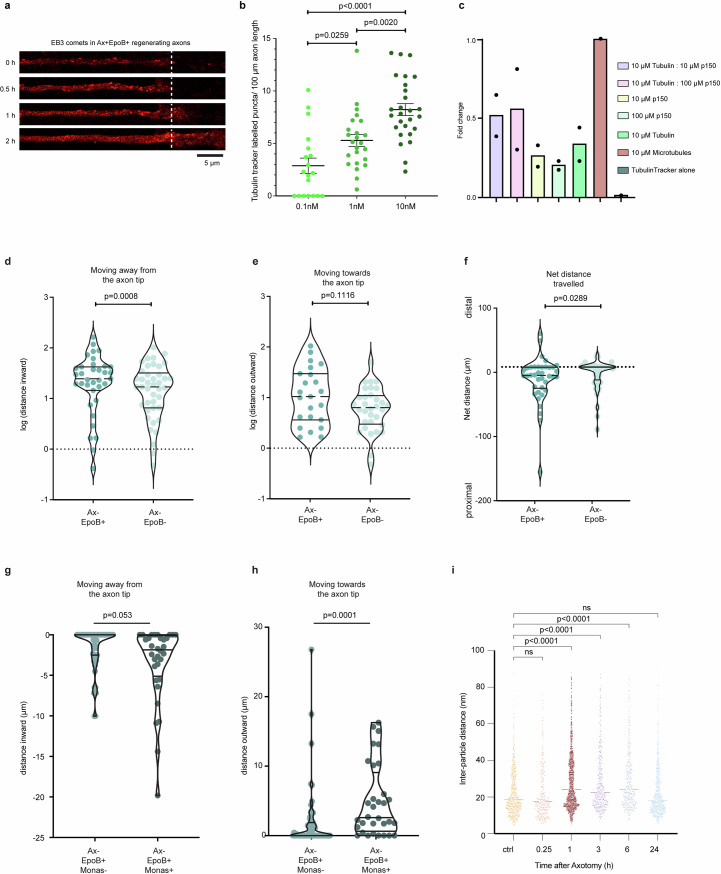
EpoB enhances microtubule dynamics and oligomer transport during regeneration. a) Live imaging of EpoB-induced regenerating thalamus axon tip transduced with EB3-mScarlet encoding lentiviruses. EB3 signals were found at the regenerating axon tip, suggesting active growth of plus-end-out microtubules. The axotomy site is shown with a white dotted line. Corresponding movies are available in Supplementary Video 7. b) Scatter dot plots showing counts of tubulin tracker puncta in thalamus control axons in the presence of different concentrations of EpoB. Mean is indicated as horizontal line. One-Way Anova was used for statistical testing, and p-values are 0.0259 (0.1 nM vs 1 nM), <0.0001 (0.1 nM vs 10 nM) and 0.0020 (1 nM vs 10 nM). N = 19 (0.1 nM EpoB), N = 24 (1 nM EpoB) and N = 27 (10 nM EpoB) puncta. c) Tubulin Tracker binding affinity to tubulin monomers, polymerized microtubules and tubulin spirals. Tubulin Tracker alone does not show any fluorescence, but it can bind and fluoresce in the presence of tubulin spirals and microtubules. The data is shown as bar graphs overlaid with data points, in which the fold change in fluorescence between different experimental conditions is plotted. Tubulin spirals were induced by p150 protein. d) Distances traveled by tubulin clusters away from the axotomy site within 30 min of observation. The violin plots represent median and interquartile range. Ax−EpoB+: *n* = 39 particles (16 axons), and Ax−EpoB−: *n* = 66 particles (29 axons). Significance was tested using the two-tailed Mann-Whitney test. P-values are reported at the corresponding comparisons on the graphs. e) Distances traveled by tubulin clusters toward regenerating sites within 30 min of observation. The violin plots represent median with interquartile range. Ax−EpoB+: *n* = 39 particles (16 axons), and Ax−EpoB−: *n* = 66 particles (29 axons) from at least three biologically independent experiments. Significance was tested using the two-tailed Mann-Whitney test. p-values are reported at the corresponding comparisons on the graphs. f) Net distance (outward vs inward) traveled by tubulin clusters within 30 min of observation. The violin plots represent median with interquartile range. Ax−EpoB+: *n* = 39 particles (16 axons), and Ax−EpoB−: *n* = 66 particles (29 axons) from at least three independent experiments. Significance was tested using the two-tailed Mann-Whitney test. p-values are reported at the corresponding comparisons on the graphs. g) Distances traveled by tubulin clusters away from the axotomy site within 10 min of observation. The violin plots represent median with interquartile range. Ax−EpoB+Monas−: *n* = 60 particles (6 axons), and Ax−EpoB+Monas+: *n* = 32 particles (7 axons) from at least three independent experiments. Significance has been tested using the two-tailed Mann-Whitney test. p-values are reported at the corresponding comparisons on the graphs. h) Distances traveled by tubulin clusters towards the axotomy site within 10 min of observation. The violin plots represent median with interquartile range. Ax−EpoB+Monas−: *n* = 60 particles (6 axons), and Ax−EpoB+Monas+: *n* = 32 particles (7 axons) from at least three independent experiments. Significance has been tested using two-tailed Mann-Whitney test. p-values are reported at the corresponding comparisons on the graphs. i) Distribution of distances between neighboring MIPs at different times after axotomy in the presence of EpoB. The dot plots show median with interquartile range. *n* = 634 (control), 256 (t = 15 min), 2025 (t = 1 h), 401 (t = 3 h), 336 (t = 6 h), 912 (t = 24 h) particles. Median: 18.5 nm (control) 17.6 nm (15 min) 24.2 nm (1 h), 22.6 nm (3 h), 24.3 nm (6 h), 18.3 nm (24 h). At t = 15 min, the observed microtubules are located at the pre-cut site as no regeneration of microtubules are observed at this time point. At t > 1 h, all the microtubules are located at the post-cut sites. One-Way Anova was used for statistical testing and p-values are >0.9999 (control vs 0.25 h), <0.0001 (control vs 1 h), <0.0001 (control vs 3 h), <0.0001 (control vs 6 h) and >0.9999 (control vs 24 h). Source data

Taking advantage of the high contrast achieved by the absence of the plasma membrane, we performed cryo-EM analyses of the microtubule shoots at the regenerating site to gain insight into their architecture and structural mechanism. We performed a series of large-scale axotomies, in which a large number of axons were cut simultaneously within an EM grid square (Fig. 3a). We collected in situ cryo-ET data (Supplementary Video 6) and cryo-EM data suitable for high-resolution single-particle analysis (SPA) (Fig. 3a, magenta circles). Despite the extremely limited target area, typically one grid square per grid, and the resulting limited number of available microtubules for analysis, we obtained a 3D reconstruction of the microtubule shoots at 3.19 Å without applying microtubule-specific pseudohelical symmetry (Fig. 3 and Extended Data Fig. 4). Image processing showed that the microtubule shoots all displayed a 13-protofilament arrangement (Fig. 3c–e), which is a physiologically relevant assembly, in contrast to the diverse protofilament arrangements (9–16 protofilaments) observed for in vitro-assembled microtubules^28–30^. This observation suggests the presence of microtubule-associated proteins (MAPs) mediating the 13-protofilament assembly of microtubules^31–33^. Our analysis also revealed that all 1,663 microtubules displayed parallel polarities (Fig. 3b,c), such that the plus ends of all microtubules would face the distal ends of the regenerating axons, extending away from the injury sites. Corroborating these cryo-EM analyses, we have also observed the presence of EB3 comets (Extended Data Fig. 5a and Supplementary Video 7) at the tip of the regenerating axons by fluorescence microscopy, which further validates that the plus ends of the microtubules are at the regenerating end. This parallel alignment of microtubules is characteristic of axons^34–36^, indicating that the mechanisms that align microtubule polarities in axons were also acting on these EpoB-induced microtubule shoots. Together, these results indicate that the microtubule shoots displayed the characteristic physiological arrangement that is normally found in axons.

## EpoB concentrates at microtubule shoots

We then examined whether the microtubule shoots were stabilized by EpoB. Our 3D reconstruction revealed an inter-dimer distance of 83.3 Å within a protofilament, compared with 81.8 Å in unstable, dynamic microtubules and 84.0 Å in guanosine-5′-((α,β)-methyleno)triphosphate (GMPCPP)-stabilized microtubules^37^ (Fig. 3g). It has been shown previously that drug-stabilized in vitro microtubules have a stretched conformation, expanding the inter-dimer distance^24^. These indicate our in situ microtubule shoots use a stretched conformation due to drug stabilization. Indeed, although with low occupancy, we detected EpoB densities in the pocket proximal to the S9–F10 loop of β-tubulin on the inner microtubule lumen side (Fig. 3f), consistent with the location found in previous in vitro studies^22–24^. In our experiments, EpoB was used at 1 nM. Given that the cellular concentration of tubulin is generally estimated to be approximately 25 µM in non-neuronal cells^38^ and even higher in neurons^39^, the EpoB-to-tubulin ratio in our experiments was significantly (three orders of magnitude) lower than those used in prior in vitro experiments to visualize epothilone–tubulin complexes (0.2 to 0.5 mM epothilone)^22–24^. Although direct observation of the enrichment is still needed, this comparison suggests that EpoB-bound tubulin accumulates at the regeneration site to stabilize the lattice and prevent the depolymerization of microtubule shoots.

## Tubulin clusters move to the injury site

**Fig. 4:**
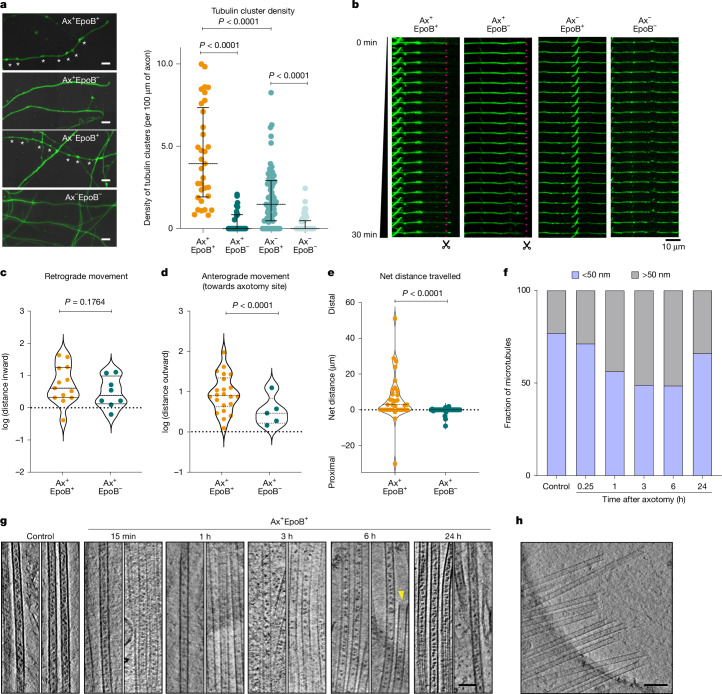
EpoB-induced tubulin cluster dynamics and microtubule maturation during axon regeneration. **a**, Regenerating axons exhibit tubulin clusters in the presence of EpoB. Left, representative images of tubulin clusters (asterisks). Scale bars, 5 µm. Right, quantification of cluster densities per 100 µm of axon shaft and tips. Ax^+^EpoB^+^: *n* = 33 axons (178 particles); Ax^+^EpoB^−^: *n* = 28 axons (16 particles); Ax^−^EpoB^+^: *n* = 69 axons (149 particles); Ax^−^EpoB^−^: *n* = 67 axons (30 particles). Scatter plots show median with interquartile range. Two-tailed Mann–Whitney test,* P* < 0.0001. **b**, Temporal snapshots showing tubulin movements with or without EpoB and axotomy. Tubulin clusters in Ax^+^EpoB^+^ axons show pronounced anterograde movement. Axotomy sites are marked with scissors and a red dotted line. Representative videos are shown in Supplementary Video 8. **c**–**e**, Analysis of tubulin cluster movements within 30 min after axotomy. **c**, Distance of tubulin clusters from the injury site (distance travelled inward). Ax^+^EpoB^+^: *n* = 33, 12 axons; Ax^+^EpoB^−^: *n* = 31, 15 axons; *P* = 0.1764 (not significant). **d**, Distance of tubulin clusters from the regenerating tip (distance travelled outward) is decreased with EpoB (*P* < 0.0001). **e**, Net displacement of tubulin clusters (distance outward versus distance inward) shows a bias for anterograde transport with EpoB (*P* < 0.0001). Data are presented as dot plot with median and interquartile range. Two-tailed Mann–Whitney test. **f**, Spatial distribution of MIPs during regeneration. Stacked bar graphs show fractions of microtubules with sparse (grey, >50 nm spacing) versus dense (blue, <50 nm spacing) MIP occupancy. Fractions of sparsely populated filaments were 23% (control, *n* = 634), 29% (15 min, *n* = 256), 44% (1 h, *n* = 2025), 51% (3 h, *n* = 401), 51% (6 h, *n* = 336); and 34% (24 h, *n* = 912). Observed microtubules were located at pre-cut sites at 15 min after axotomy and at post-cut sites at 1 h after axotomy. **g**, Cryo-ET reconstructions showing MIPs at different stages of regeneration. A yellow arrowhead indicates a MIP-free microtubule tip. Scale bar, 50 nm. A representative reconstruction is presented in Supplementary Video 6. **h**, Tomographic snapshot of regenerating axon tips with MIP-free microtubules. Scale bar, 100 nm. Source data

Next, we examined the origin of the microtubule shoots. We initially hypothesized that de novo synthesis of tubulin subunits can fuel microtubule polymerization at the regenerating site, possibly similar to our previous cryo-ET observation indicating local actin synthesis at axon branching points^40^. However, we ruled out the possibility that tubulin subunits themselves are locally synthesized, as ribosomes were not readily visible at the injury sites (Fig. 2i and Supplementary Video 6). Fluorescence live-cell imaging of microtubules using the Tubulin Tracker dye revealed the formation of bright puncta upon addition of EpoB (Fig. 4a, asterisks) irrespective of whether axotomy was performed. The density of the puncta increased in a dose-dependent manner (Extended Data Fig. 5b). These puncta moved bi-directionally along axons (Fig. 4b–e, Extended Data Fig. 5b–f and Supplementary Video 8), in contrast to sparse puncta in untreated axons (Fig. 4a,b). Therefore, we reasoned that these bright puncta could be clusters of short microtubules or tubulin oligomers that can be transported towards the tip of the regenerating site, as we validated that Tubulin Tracker similarly detects tubulin oligomers and microtubules in our control experiment (Extended Data Fig. 5c). Following axotomy, a substantial proportion of EpoB-induced clusters exhibited anterograde movement towards the injury site (Fig. 4b–e and Supplementary Video 8), with 39% travelling more than 5 μm and 9% moving retrogradely. By contrast, clusters in the absence of EpoB showed minimal displacement, with the majority (74%) remaining within 1 μm and 16% moving retrogradely (Fig. 4b–e and Supplementary Video 8). This observation suggests that once an injury occurs, a signal is propagated towards upstream of the injury site, triggering anterograde transport of tubulin clusters. We further investigated whether the transport of the clusters is mediated by motor proteins. Kinesin-5 (KIF11 in humans) has been previously shown to limit transport of short microtubules by other motor proteins^41^. Indeed, when we inhibited kinesin-5 using monastrol in our EpoB-treated axons, there was an increased transport frequency of tubulin clusters (Extended Data Fig. 5g,h and Supplementary Video 9), supporting a role of kinesin-5 as a negative regulator of the process. Furthermore, we validated the colocalization of the tubulin clusters with kinesin motors (Supplementary Video 10). These results together indicate that the transport is driven by an anterograde kinesin-based molecular motor.

## Tubulin spirals drive microtubule regrowth

**Extended Data Fig. 6:**
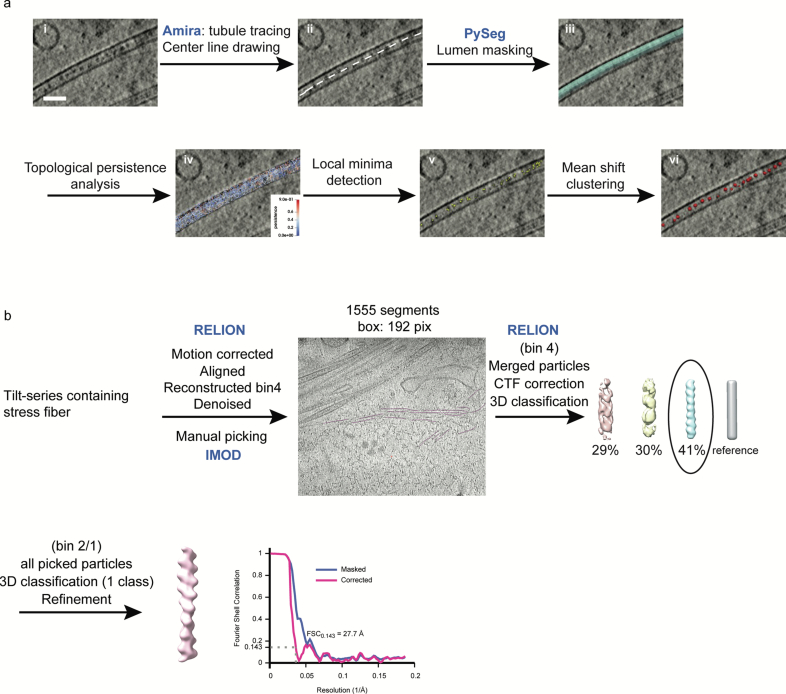
Workflows for MIP analysis and stress-fiber subtomogram averaging. a) Processing workflow for the MIP analysis. i) Zoomed-in view of a MT in a 2D slice of a tomogram, scale bar: 50 nm. ii) The centerline is traced by Amira. iii) The lumen is segmented by applying distance transform on the centerline. iv) PySeg analysis extracting the local minima and their connections in the MT lumen and MT shell. The color bar represents the topological persistence, the metric used to rank the minima relevance. The information of the shell is used to determine the threshold for the lumen. v) Thresholded local minima by persistence in the MT lumen. vi) MIP obtained by applying mean shift clustering to v. b) Processing workflow for stress-fiber subtomogram averaging. Tilt-series alignment and tomographic reconstructions were performed within RELION 5.0. A total of 1555 segments of filaments were manually selected from the area of interest using IMOD, and the corresponding coordinates were imported into RELION for subtomogram extraction in the form of a 2D stack. For subtomogram averaging, a cylinder reference was used, followed by 3D classification to create an initial model, and further refinement was performed using a combination of classification and refinement schemes without applying the actin helical symmetry. The nominal resolution was 27.7 Å. CTF correction was applied only during subtomographic analysis, following 3D reconstruction using RELION 5.0.

To further understand the provenance of the microtubule shoots, we took advantage of the presence of microtubule inner-luminal proteins (MIPs). MIPs are tightly packed within microtubules, with neighbouring particles spaced 18.5 nm apart in control neurons (Fig. 4f,g and Extended Data Fig. 5i), and primarily consist of MAP6^42^ and, potentially, tubulin chaperones^43^. They are commonly found inside neuronal microtubules, regardless of species or neuron type. Our tomographic data revealed that microtubule shoots at the regenerating tip often lack MIPs. This may reflect rapid microtubule polymerization at the injury site and delayed MIP incorporation. To investigate further, we collected tomographic data at the pre-cut site 15 min after axotomy, before microtubule shoots appeared but after the initial membrane closure, and found that the particle occupancies in microtubules treated with EpoB were similar to those in control microtubules (Fig. 4f,g). We then quantified MIP occupancy at various time points during regeneration (Fig. 4f and Extended Data Fig. 6). Our analysis revealed the presence of microtubule shoots with varying levels of MIP densities, which decreased at the 1 h time point after axotomy (Fig. 4f,g). In extreme cases, microtubules observed at the remote distal ends were found completely without MIPs (Fig. 4g,h, yellow arrowhead). The proportions of microtubules with an inter-MIPs distance of more than 50 nm (indicating sparse MIPs presence) were 23% (control), 29% (15 min), 44% (1 h), 51% (3 h), 51% (6 h) and 34% (24 h) (Fig. 4f). More MIPs appeared as repair progressed, eventually returning to an occupancy resembling that in the control, suggesting the maturation of newly generated microtubules. Together with the presence of EB3 at the regenerating tip (Extended Data Fig. 5a and Supplementary Video 7), we concluded that microtubule polymerization is activated at the regenerating site to form microtubule shoots rapidly. Established microtubules may also be transported from the axon shaft as a standard mode of axonal transport^41^.

**Fig. 5:**
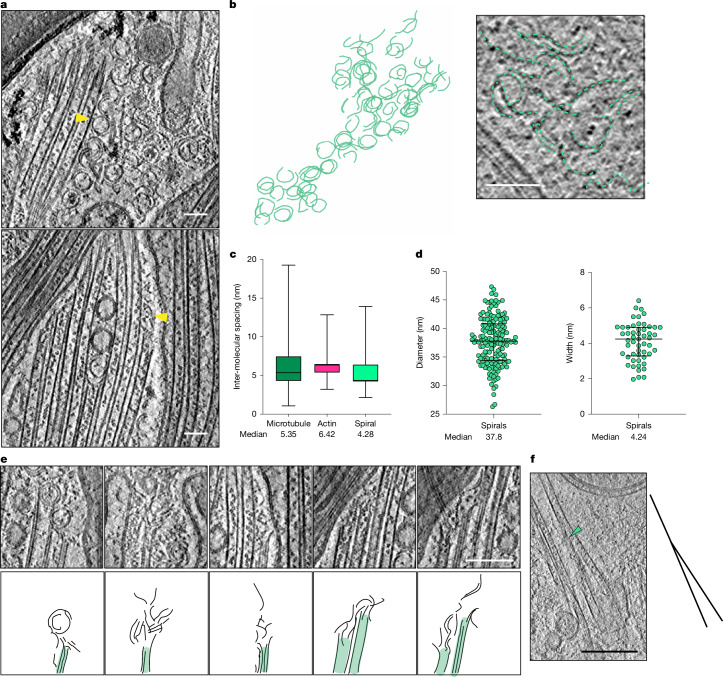
Spiral-like tubulin oligomers as precursors of polymerizing microtubules. **a**, Snapshots of cryo-ET sections showing clusters of spiral-like tubulin oligomers (yellow arrowheads) at post-cut sites. Scale bars, 50 nm. The corresponding tomogram is shown in Supplementary Video 11. **b**, Segmentation of tubulin spirals from a tomographic reconstruction in 2 different views (80° rotated) to visualize the geometry of the spiral structures. Right image highlights oligomers with loose packing. Scale bar, 50 nm. **c**, Intermolecular spacing of microtubules, F-actin filaments and tubulin spirals. In box plots, the centre line represents the median, box edges delineate 25th and 75th percentiles, and whiskers extend from minimum to maximum values. *n* = 170 segments (6 microtubules), *n* = 97 segments (8 F-actin filaments), *n* = 182 segments (16 tubulin spirals). **d**, Size distribution of spiral-like oligomers. Dot plots show the distribution of diameter and width of tubulin oligomers with median and 25th and 75th percentiles as horizontal lines. Left, diameter: *n* = 163 oligomers, median: 37.8 nm, 25th percentile: 34.3 nm, 75th percentile: 40.7 nm. Right, width: *n* = 53 oligomers, median: 4.2 nm, 25th percentile: 3.2 nm, 75th percentile: 4.9 nm. **e**, Top, magnified images of tomographic sections, highlighting tubulin spirals attached to microtubule ends. Some spirals are unfolding into straighter conformations. Bottom, outline sketches of the polymerizing microtubules (green) with oligomers attached. Scale bar, 50 nm. **f**, Tomographic snapshot showing microtubule branching (green arrowhead) with a schematic on the right. Scale bar, 200 nm. The corresponding tomogram is shown in Supplementary Video 12. Source data

The notion that microtubule shoots polymerize at the distal end suggests that the observed tubulin clusters that were transported anterogradely in the presence of EpoB (Fig. 4 and Supplementary Video 8) are likely to represent tubulin oligomers. Previous studies have shown that tubulin oligomers form an open spiral assembly from longitudinally connected tubulins^44–47^, exposing the luminal surface outwards. This formation is also observed when Taxol-stabilized microtubules disassemble into tubulin spiral oligomers under depolymerizing conditions^48^. The binding sites for Taxol and EpoB are located on the luminal side of β-tubulin and they share a similar stabilization mechanism. Therefore, we reasoned that these stabilized tubulin oligomers might originate from the depolymerization of EpoB-stabilized microtubules. The oligomers could serve as a readily available pool of tubulin subunits that could be quickly incorporated into new microtubules at the regeneration site. We searched our cryo-ET data for tubulin clusters or oligomers that might act as precursors for rapidly growing microtubule shoots in proximity to the regenerating portion of axons. We found oligomers (Fig. 5a,b and Supplementary Video 11) with an intermolecular spacing of 4.6 nm (Fig. 5c, compared with 5.5 nm for the measurement of F-actin as a control), a diameter of 37.8 nm (Fig. 5d, left) and a width of 4.2 nm (Fig. 5d, right), consistent with the dimensions of a tubulin monomer (4 nm × 5 nm) and the diameter of single-protofilament tubulin spiral oligomers assembled in vitro^48^ (38.6 nm). These oligomers exhibited spiral features with variable degrees of packing, ranging from tightly coiled structures to fully extended, linear string-like forms (Fig. 5b). They frequently clustered together, occupying a large volume within the cytosol that were not enclosed by lipid membranes. The oligomers were attached to the tips of microtubules (Fig. 5e), unwound from the common spiral-like morphology, consistent with the in vitro observation of purified tubulins^45,46^, suggesting that we have captured microtubule polymerization in situ by cryo-ET. Reinforcing the possibility that microtubules are locally assembled, we found several deformed microtubules—for example, branching morphologies or doublet-like protofilament arrangements—at regenerating sites treated with EpoB (Fig. 5f and Supplementary Video 12). A mother microtubule with splayed or spread protofilaments emerging into new daughter microtubules was identified, which presumably represented an intermediate in the microtubule assembly process (Fig. 5f and Supplementary Video 12). We have previously observed such a specific microtubule assembly in the presence of the microtubule-nucleation factor SSNA1 in vitro^49^. We interpret our finding as the urgent need for microtubule polymerization resulting in fast and deformed growth, which is then repaired upon the normalization of neuronal homeostasis.

## Cargo transport to regeneration sites

**Extended Data Fig. 7:**
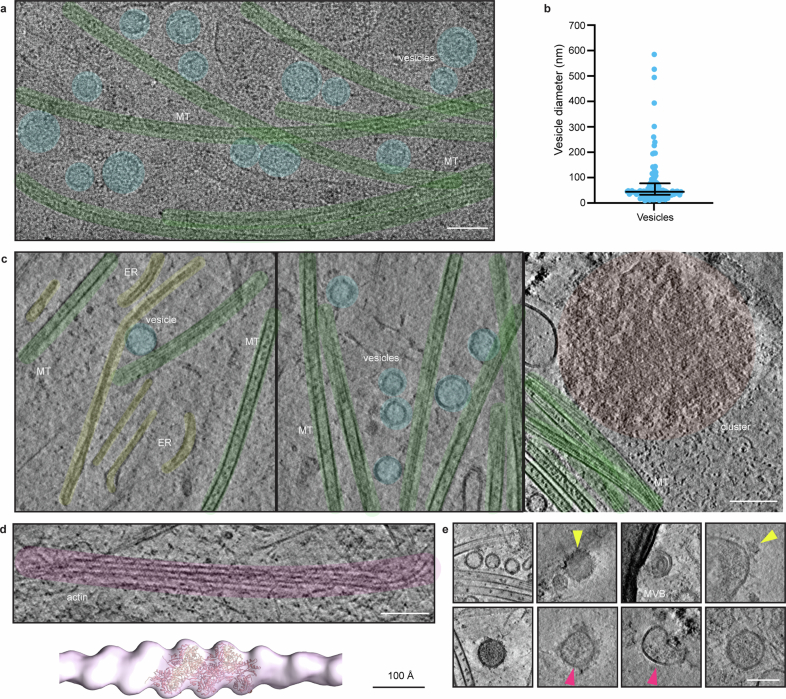
Cellular and vesicular components at regenerating axon sites. a) Representative cryo-EM snapshot of cellular components at regenerating sites after axotomy in the presence of EpoB. Membranous and vesicular entities accumulate at regeneration sites. Vesicles are highlighted in light blue and microtubules (MT) in green. Scale bar: 100 nm. b) Vesicle size distribution as dot plot. *n* = 110 vesicles, median: 43.9 nm, 25th percentile: 32.7 nm, 75^th^ percentile: 76.6 nm. c) Representative sections of a cryo-ET reconstructions of a regeneration site showing endoplasmic reticulum (ER) membranes (highlighted in light yellow), presynaptic vesicles (highlighted in light blue), and clusters of materials (highlighted in light brown) near microtubules. Scale bar: 100 nm. d) Cryo-ET section showing actin stress fiber formation (highlighted in light pink). Scale bar: 100 nm. Subtomogram average of actin fibers at 27.7 Å resolution for validation. e) Various cryo-EM snapshots of regenerating axons showing vesicles coated with proteins (yellow arrowheads) and vesicles with components in inner compartments (magenta arrowheads). Scale bar: 100 nm. Multivesicular body (MVB). Source data

Cryo-EM-based observations and tomographic reconstructions of EpoB-mediated regenerating axons revealed the presence of various cellular components (Extended Data Fig. 7a–c). Actin stress fibres formed along the microtubule shoots (Extended Data Fig. 7d). We found that vesicles transported along the microtubule shoots and accumulated at the microtubule tips (Fig. 2i). The surrounding vesicles ranged up to 600 nm in diameter (Extended Data Fig. 7b) with a large population of small vesicles (median 44 nm; Extended Data Fig. 7b,c), presumably presynaptic vesicles^50^. We observed endoplasmic reticulum stretching towards the tip of the regenerating axon as well as clustered materials that were not enclosed by a membrane bilayer (Extended Data Fig. 7c). Various dense materials of different sizes were found inside of vesicles or on their membranes (Extended Data Fig. 7e).

**Fig. 6:**
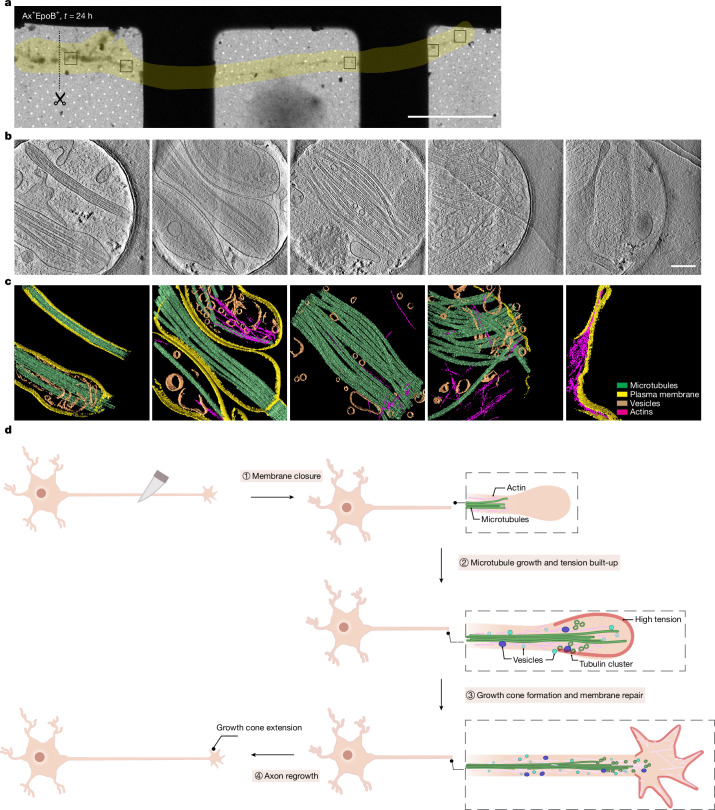
Structural visualization and schematic sequence of EpoB-driven axon regeneration. **a**, Cryo-EM image (grid view) of a regenerating axon in the presence of EpoB, 24 h after axotomy. The axotomy site is indicated by a black dotted line. The yellow shading highlights the path of the regenerating axon. Black boxes indicate positions at which cryo-ET data were collected and reconstructed. Sections are shown in **b**,**c**. Scale bar, 50 µm. **b**, Cryo-ET snapshots of a regenerating axon 24 h after axotomy in the presence of EpoB, from the boxed areas in **a**. Scale bar, 200 nm. **c**, Segmentation of the cryo-ET reconstructions shown in **b**. Some areas are surrounded by plasma membranes and some remain uncovered. Growth cones are established at the regenerating axon tip at this time. **d**, Summary of the molecular events in EpoB-induced axon regeneration. (1) The initial reaction after axon injury or axotomy is membrane closure, resembling the morphology of a retraction bulb. (2) In the presence of EpoB, stabilized microtubules shoot out from the injury site towards the plasma membrane, resulting in increased membrane tension. Using these stabilized microtubules as tracks, tubulin clusters and vesicles as membrane source are shuttled towards the growing axon. (3) Vesicles deliver materials for regeneration, including the necessary membrane support at the regenerating front, resulting in normalizing membrane tension (4). Ultimately, repair is complete, and a new growth cone is established at the axon tip that navigates the growth of the axon.

**Extended Data Fig. 8:**
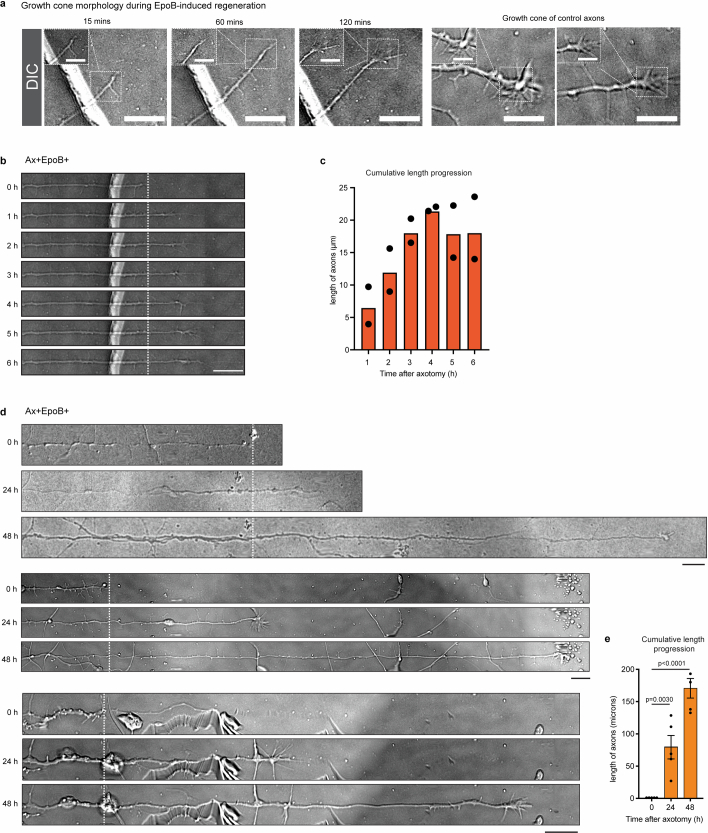
Dynamic progression of EpoB-induced axon regeneration over hours. a) Live images of an EpoB-induced, regenerating axon tip shown at different time points during repair. For about 2 h, the regenerating front displays a flat rapidly moving axon tip. After two hours, a typical actin-rich finger-like growth cone appears. Scale bar: 10 µm. Zoomed-in images of the tip are shown in the insets. Scale bar: 5 µm. b) Live images of EpoB-induced regenerating axons recorded continuously for 6 h. Straightened axon snapshots at different time points are shown for comparison with the axotomy site indicating with a dotted white line. Scale bar: 10 µm. c) Quantification of the cumulative length of axons during EpoB-induced regeneration over 6 h. *n* = 2 axons from two biologically independent experiments. The bar graph is overlaid with corresponding data points. d) Snapshots of EpoB-induced regenerating axons captured at 0, 24 and 48 h. Images were straightened for comparison. EpoB-induced axonal growth continues for at least 48 h beyond axotomy. Scale bar: 10 µm. e) Quantification of the cumulative length of axons during EpoB-induced regeneration. The bar graph shows mean ± s.e.m. Significance was tested using One way ANOVA. P-values are 0.0030 (0 h vs 24 h) and <0.0001 (0 h vs 48 h). *n* = 5 axons from three biologically independent experiments. Source data

Live imaging of regenerating axons revealed the initial rapid regrowth of the axon, which then slowed down (Fig. 2h and Extended Data Fig. 8). With no obvious actin growth cone appearing during the initial stage of regeneration, the tip exhibited a smooth and flat regeneration front. At 2–4 h, several membrane protrusions prototypical of growth cone morphology appeared (Extended Data Fig. 8a). During this time, the regenerating axon tip experienced the recovery of the membrane tension (Fig. 2d) until around 4 h. Within 6 h, the regenerating axon did not visibly grow (Extended Data Fig. 8b,c), but after the 24 and 48 h time points, these axons grew extensively (Extended Data Fig. 8d,e). Cryo-EM images of regenerating axons at 3 h and 6 h after axotomy showed abundant numbers of vesicles (Extended Data Fig. 2b,c). Patches of membranes appeared sporadically alongside the axon, probably stabilized through crosslinking to cellular components. Membranes were found to surround actin stress fibres (Extended Data Fig. 2c(ii)). Twenty-four hours after axotomy, regenerated axons re-established their typical morphology (Fig. 6a–c), topped with an actin-based growth cone (Fig. 6c, right).

Collectively, our analysis demonstrated that axonal repair was triggered by rapid microtubule growth induced by the stabilization of the lattice by EpoB. Without an actin network at the regenerating site, the growing microtubules apply mechanical force on the sealed plasma membrane, increasing membrane tension. This is followed by bulk intracellular transport along the microtubule shoots, suggesting that these vesicles are probable sources of the newly generated plasma membrane, and membrane tension normalizes within 4 h after injury. Recovery of the axon growth cone is followed by the regrowth of the axon observed at 24 h to 48 h time points, leading to the subsequent slow rebuilding process of the neural network, which takes weeks^20^.

## Discussion

The capacity of axon regeneration is influenced by a number of factors, including neuron type, developmental stage, age of the affected neuron and the extent of damage and the surrounding environment. In the CNS, regeneration is particularly hindered by a non-permissive microenvironment characterized by inhibitory molecules, lack of trophic support and the presence of oligodendrocytes^51^. However, removing external inhibitory cues is not sufficient to activate CNS axon regeneration. Although CNS neurons have shown the ability to regrow in a permissive peripheral nervous system environment^52^, their functional recovery remains limited^53^. For example, only a small subset of retinal ganglion cells regenerate within a peripheral nervous system graft^53^. These observations indicate the criticalness of intrinsic axonal properties for successful CNS regeneration, independent of external inhibitory factors.

In this study, we developed a cryo-EM–cryo-ET pipeline to induce axonal injury and monitor the intrinsic axonal response at molecular resolution. Using the FDA-approved anti-cancer drug EpoB, which has demonstrated beneficial effects during recovery from spinal cord injuries in mice^20^, we triggered axonal regeneration in CNS neurons. Following injury, EpoB-treated neurons switch to a pro-regenerative state despite disrupted cellular homeostasis. We found that rapid axon regeneration is driven by EpoB-stabilized microtubule shoots, and not by a growth cone. These microtubules mechanically push the plasma membrane plug that forms as an immediate contingency after injury. We determined that axon regeneration proceeds through four phases (Fig. 6d): (1) membrane closure and the formation of a retraction bulb; (2) rapid microtubule growth; (3) recovery of membrane integrity and re-establishment of the growth cone; and (4) axon regrowth. Axonal injury and mechanical damage to the plasma membrane trigger a Ca^2+^ influx by activation of Ca^2+^ channels^7^ and induce microtubule depolymerization, causing axon retraction and the formation of a retraction bulb once the membrane is resealed (phase 1). During EpoB-mediated regeneration, rapid microtubule growth stretches the membrane plug as tubulin clusters are delivered to the injury site, a process that would only be possible with the aid of EpoB or similar microtubule-stabilizing factors in the presence of a Ca^2+^ pool at the injury site (phase 2). We propose that EpoB concentrates at tubulin oligomer clusters, which are transported to the regenerating tip to support rapid microtubule extension. Similar tubulin oligomers have been observed in vitro in the presence of MAPs, nucleotide analogues and Taxol^44–48^, suggesting that EpoB may similarly promote their formation. In addition to facilitating oligomer assembly, EpoB further stabilizes the microtubule lattice at the shoots, thereby promoting regeneration. Notably, although the local accumulation of EpoB at the regeneration site could result from activated anterograde transport (Fig. 4b–e, Extended Data Fig. 6g,h and Supplementary Videos 9 and 10), EpoB may also become enriched on newly polymerizing microtubules throughout the axon, where it acts to stabilize dynamic microtubules regardless of position. Overall, regeneration growth plateaus approximately 2–3 h after axotomy, probably owing to mechanical limitations from the membrane stretching and depletion of materials. At this point, microtubule shoots may serve as scaffolds and transportation routes, while lipid replenishment occurs more gradually (phase 3), and finally the steady growth of the axon resumes (phase 4).

Several types of vesicles and cellular materials are actively transported along microtubules to re-establish axonal identity and integrity. This activation indicates that information of axon injury is transmitted upstream, potentially to the soma^54–57^. The underlying mechanism that drives the sequence of events remains unknown. Future studies will be necessary to determine how neurons regulate and control axonal repair and the sequence and type of intercommunication between the injury site and the soma.

Although we successfully elucidated the intracellular mechanism underlying axon regeneration, our study remains limited in addressing the extracellular context that influences neural repair. It should be noted that EpoB has also been implicated in scar repair^20^, suggesting that its effects extend beyond intracellular microtubule stabilization on the injured axons. Extrinsic factors such as tissue stiffness^58^, inhibitory and guidance molecules^59^, injury location within the brain or nervous system, and interactions with other cells such as glia cells have roles in determining regenerative success^60^. Future development of the cryo-EM visualization platform will be advantageous to directly visualize the interplay between the extracellular environment and intracellular repair mechanisms at the molecular level, in particular, how extracellular cues influence microtubule remodelling, growth cone formation and axon extension. Understanding the interactions at the molecular level will also be beneficial for designing more effective therapeutic strategies that not only target intracellular regeneration but also optimize the surrounding microenvironment to enhance neural recovery.

Overall, our study highlights an unexpected molecular response to EpoB that activates neuronal regeneration capabilities. EpoB alters the sequence of events following injury and we found that neuronal cells can adapt to the disruption of the order of cellular events until normal homeostasis is recovered. Our study also provides fundamental molecular insights into the general mechanisms underlying neuronal regulation, demonstrating the plasticity of neurons in accommodating homeostatic disruptions. These disruptions include the absence of a growth cone during neurite regrowth, alternation of the membrane tension and hyper microtubule formation in a confined area. Understanding the basis of axon regeneration is critical for improving therapeutic options and recovery after traumatic axonal injury.

## Methods

### Grid preparation

Quantifoil gold grids (R1/4, 200 mesh, 100 Holey SiO_2_ Films or Multi A, 300 mesh) were glow discharged using a Pelco easiGlow at 15 mA current and 0.38 mbar residual air pressure for 60 s. Then grids were immediately placed in a cell culture dish (Greiner Bio-One 627170), covered with 2 ml of 1 mg ml^−1^ poly-l-lysine (Sigma Aldrich P2636) prepared in 0.1 M borate buffer (di-sodium tetraborate decahydrate, Carl Roth 1303-96-4) and incubated overnight at 37 °C. Afterwards, grids were thoroughly washed three times in 1× PBS (Gibco 10010049) or ultrapure water and coated with 5 µg ml^−1^ laminin (Gibco 23017015) solution for at least 2 h at 37 °C. After laminin coating, grids were covered with Neurobasal medium (Gibco 21103049) and incubated at 37 °C until cells were seeded.

### Preparation of primary mouse thalamus explants and dissociated neurons

All animal procedures were approved by the Animal Care and Use Committee (ACUC) of the National Heart, Lung and Blood Institute (NHLBI) animal protocol H-0331 in accordance with NIH research guidelines for the care and use of laboratory animals. Cultures were prepared from embryos (E15.5) of CD-1 mice (Charles River Laboratories). Thalamus tissue was dissected from the brain of embryos and placed in cold Hanks’ Balanced Salt Solution (HBSS, Gibco 14170112) supplemented with 1× HEPES (Gibco 15630080). The thalami were then cut into smaller fragments. For explant cultures, the thalamus tissue with a size of approximately 200 µm was placed onto coated μ-Dish 35 mm (Ibidi 80156), glass coverslips, or coated electron microscopy (EM) grids (see grid preparation above). For dissociated cells, thalamus pieces were incubated with 1× Trypsin-EDTA (0.5%, Gibco 15400054) and 1× DNase for 15 min at 37 °C. After the enzymatic treatment, tissue pieces were washed three times with plating medium (Neurobasal medium containing 5% fetal bovine serum (Gibco 10437028), 1× B27 (Gibco 17504044), 1× Glutamax (Gibco 35050061)) and then dissociated by trituration in 2 ml of plating medium. Cells were seeded onto coated Ibidi petri dishes or engraved glass coverslips (Electron Microscopy Sciences, 72264-23) at a concentration of 150,000 cells per ml. After 1 h of plating, the medium was exchanged to 1× Neurobasal supplemented with 1× B27, 1× penicillin-streptomycin (Gibco 15140122), and 1× Glutamax. After 3 days in vitro (DIV 3), half of the culture medium was exchanged with fresh medium.

### Distal axotomy and induction of axon regeneration

Micropipettes used for axotomy were made of borosilicate capillaries (inner diameter = 0.0056, outer diameter = 0.022; Drummond Scientific Company 1-000-005) and were formed using a Micropipette puller P-97 (Sutter Instrument) with a final tip diameter of ~1 µm. A freshly prepared micropipette was placed into a needle holder of a micromanipulator (Eppendorf TransferMan 4r) mounted on an inverted Leica DMi8 microscope. Distal axotomy was performed on thalamus explants on DIV 6–9 as described above. Approximately 5 axons were identified using a 5× air objective and visualized with a 40× air objective. Lesions were performed at least 250 µm away from the centre of the explant by swiftly moving the tip of the micropipette across the axons. The distal part of the axon after the injury was gently but thoroughly cleared near the cut site. Performing axotomy on different surfaces, such as glass and EM grids, requires adjustments in handling. On a glass surface, axotomy is straightforward due to the rigidity and stability of the substrate. However, when working with EM grids, which are more delicate and fragile, greater precision and care were required to avoid damaging the grid surface. We found that the grid surface remained visibly intact when the cut was clean, and the procedure was reproducible giving consistent experimental outcomes. Axonal regeneration was induced by adding 1 nM of EpoB (from *Sorangium cellulosum*; Sigma E2656) except for control and comparison experiments. EpoB was dissolved in DMSO (Sigma D8418) at a concentration of 2 mM and stored at −20 °C. For comparison, 1 nM Taxol (Sigma Aldrich 1912) was also used for inducing axon regeneration (Extended Data Fig. 1c). Taxol was dissolved in DMSO at 1 mM concentration and stored at −20 °C.

### Cryo-EM sample preparation

Mouse primary thalamus explant tissue was plated onto coated cryo-EM grids (see above) and cultured for 6–9 days. The grid squares on the edge of the grid were gently pierced by a sharp tweezer, so that the broken grid squares could be used as a reference point to find axon cut sites. Phase contrast images of the grids were then captured with a 4× objective using an EVOS digital colour fluorescence microscope (Thermo Fisher Scientific). The thalamus explant tissues were then subjected to axotomy as described before. Immediately after axotomy, the culture dishes were either treated with vehicle control DMSO or 1 nM of EpoB for different times. At the given time point, grids (control and EpoB-treated) were drained of excess liquid by blotting from the side with filter paper. Then, immediately 3 µl of conditioned neurobasal medium (medium incubated with untreated neurons for the same number of days in vitro) was added to the grids. For hypotonic experiments, neurons were incubated with hypotonic solution (culture medium at DIV 8 diluted 3 times with ultrapure water) for 5 min before vitrification. The grids were then vitrified in liquid ethane using a Vitrobot Mark VI Freeze-plunger (Thermo Fisher Scientific), conditioned at 25 °C and 100% humidity. The grids were screened on a Glacios cryo-TEM operated at 200 kV and equipped with a Falcon 4 direct electron detector (Thermo Fisher Scientific) for determining ice thickness.

### Lentivirus production and transduction

Lentiviral plasmids containing genes of interest and tagged with fluorescent proteins for labelling neuronal organelles were obtained as follows. pTRIP-CMV-EB3-mScarlet-I was constructed via Gibson assembly of EB3-mScarlet into the pTRIP-CMV-GFP-2A vector opened at the NheI and KpnI sites. eGFP-KIF5A was a gift from J. Bonifacino (Addgene plasmid 172201)^61^. pBa.GFP-KIF5B was a gift from M. Bentley (Addgene plasmid #134625)^62^. pGFP-Kif5c was a gift from M. Peckham (Addgene plasmid #71853)^63^. For overexpression of kinesin genes, we constructed the lentiviral target vector pLEX-hSYN-mCherry from a pLKO.1 backbone by removing the U6 shRNA overexpression cassette and replacing the CMV promoter with the neuron-specific human synapsin promoter by Gibson Assembly. All kinesin genes were amplified by PCR and subcloned into pLEX-hSYN-mCherry by Gibson Assembly. Lentiviral particles were produced as described previously^49^. Lentiviral vectors with viral packaging vectors (psPAX2 and pCMV-VSV-G) were co-transfected in Lenti-X-293T cells (TaKaRa) using TransIT-293 transfection reagent (Mirus Bio 2700). The medium was exchanged to Neurobasal medium the next day and incubated with cells for 24 h. The virus-containing medium was then passed through a 0.45-µm filter, aliquoted, and stored at −80 °C. Alternatively, lentiviruses were concentrated using Lenti-X-concentrator (TaKaRa 631231) according to the manufacturer’s protocol. The amount of virus used for experiments was determined by adding different volumes of virus to neurons and visually inspecting the expression levels of fluorescent protein. After 1 h of plating, neurons were transduced with the desired lentiviruses on DIV 0, and the medium was exchanged the next day. On DIV 8–10, live imaging was performed as described below.

### Live-cell imaging and analysis

Primary neurons or tissue samples were cultured on µ-Dish 35 mm (ibidi 80136) or µ-Dish 35 mm with location grids (ibidi 80156) for 6–10 DIV at 37 °C and 5% CO_2_. For the tracking of tubulin, neurons were incubated with 100 nM Tubulin Tracker Green (Thermo Fisher Scientific T34075) for 15−30 min or with 100 nM SiR-tubulin (Cytoskeleton Spirochrome CY-SC002) for 30 min, respectively. Neurons were gently washed once, and 1 ml of conditioned neurobasal medium was added to the dish. The movements of fluorescently labelled spots inside the axons were recorded using a Nikon A1R microscope or a Zeiss LSM 980 Airyscan microscope, with a 60× oil-immersion objective and a stage top incubator set to 37 °C and 5% CO_2_. The microscope was controlled using NIS-ELEMENTS software (Nikon) or Zeiss ZEN software. Movies were recorded immediately after distal axotomy at time intervals of 5 min and 30 s for SiR-tubulin and Tubulin Tracker, respectively. For Supplementary Video 10, the movements of Tubulin Tracker and kinesins were captured every 5 s. The fluorescence tracking and movement analysis was done with FIJI/IMAGEJ 2.3.0/1.53f^64^ and the KymoToolbox plugin according to the available protocol^65^. It should be noted that Tubulin Tracker might also affect microtubule stabilization; however, these effects appeared to be minimal, as we observed clear differences between axons treated with and without EpoB. For imaging EB3 dynamics (Extended Data Fig. 5a), thalamus neurons were transduced with lentivirus encoding EB3-mScarlet-I on DIV 5. On DIV 8, axotomy was performed, and EpoB-induced regenerating axons were imaged with a ZEISS LSM 980 Airyscan microscope using a 60× oil-immersion objective at different time points. Images were acquired every 4 s for 2 min at specified time points. For colocalization of microtubules and plasma membrane, thalamus explants were incubated with 100 nM SiR-tubulin for 1 h, subjected to axotomy, and allowed to regenerate in the presence of 1 nM EpoB. After 1 h of regeneration, 0.1× final concentration of CellMask (ThermoFisher Scientific C10046) was added to the regenerating axons, and the regenerating front was immediately imaged using a Nikon A1R confocal microscope with a 60× oil-immersion objective. For Extended Data Fig. 3c, thalamus explants after axotomy were incubated with fluorescently labeled tubulin antibody (1:500) and imaged for 30 min to directly visualize any potential microtubules. We did not observe any microtubule staining, confirming that the membrane was sealed. For imaging actin, microtubules, and plasma membrane together (Extended Data Fig. 1f), 50 nM of SPY555-FastAct (Cytoskeleton Spirochrome CY-SC205), 50 nM of SPY650-Tubulin (CY-SC503), and a MemGlow 488 (1:200,000) (Cytoskeleton MG01-10) were incubated with the neurons for 30 min prior to imaging.

### Immunofluorescence analysis

Thalami explants cultured on glass coverslips at DIV 6 were subjected to axotomy and treated with either DMSO or 1 nM EpoB, followed by fixation for 15 min with 4% paraformaldehyde (Electron Microscopy Sciences 15710). Paraformaldehyde-fixed explants were permeabilized with 0.5% Triton X-100 (Sigma Aldrich T9284) prepared in 1× PBS for 5 min at room temperature. Explants were then blocked in 10% Normal Goat Serum (Cell Signaling Technology 5425) for 1 h and stained with anti-MAP2 (Millipore AB5622, 1:1,000) and anti-Tau1 (Millipore MAB3420, 1:1,000). The samples were washed with 1× PBSTx (PBS containing 0.1% Triton X-100) and incubated for 1 h with donkey anti-mouse Alexa Fluor 647 (ThermoFisher Scientific A31571, 1:1,000) and goat anti-rabbit Alexa Fluor 568 (ThermoFisher Scientific A11036, 1:1,000). Nuclei were stained with Hoechst 33342 (0.02 μg ml^−1^, ThermoFisher Scientific 62249). For the assessment of membrane integrity (Extended Data Fig. 1d), explants were processed without permeabilization and Triton X-100, and stained with 12G10 (anti-α-tubulin, DSHB AB_1157911, 1:500) and CellBrite Red (Biotium 30023, 1:5,000) for plasma membrane staining. Cells were mounted using ProLong Gold anti-fade (ThermoFisher Scientific P36934). Cells were imaged using Zeiss LSM 880 or Zeiss LSM 780 confocal microscopes with the tile scan option with 60× and 40× objectives. Acquired images were processed using FIJI.

### Polygon montage of cryo-EM images

Polygon-montage images covering a whole grid square, in which distal axotomy was performed, were acquired on a Glacios cryo-TEM operated at 200 kV, equipped with a Falcon 4 direct electron detector at a magnification of 13,500×, at binning 4, corresponding to a pixel size of 4.121 nm at a defocus of −200 µm. Polygon montages for Extended Data Fig. 3 were acquired using Titan Krios cryo-TEM (ThermoFisher Scientific) equipped with a Gatan Bioquantum energy filter and a K3 Summit direct detector at an acceleration voltage of 300 kV using SerialEM^66^ at a magnification of 19,500×, at binning 2, corresponding to a pixel size of 0.9 nm at a defocus of −30 µm. Images were binned 4× to reduce file size and facilitate processing.

### Cryo-ET data acquisition

Low-magnification (13,500×) images of the grids were recorded and manually matched to previously recorded phase contrast images. Broken grid squares were used as reference points to find axon cut sites. Locations proximal to the axon cut site as well as further away to visualize actively regenerating axons were selected. Tomographic data were acquired using a Titan Krios TEM equipped with a Bioquantum energy filter and a K3 Summit direct (Gatan) detector at an acceleration voltage of 300 kV. Images were recorded at a nominal magnification of 33,000×, corresponding to a calibrated pixel size of 2.67 Å.

Tilt series were collected in super-resolution mode using SerialEM^66^, ranging from −60° to 60° with 2° angular increments, yielding a total of 61 images per tilt series. The total accumulated electron dose per tomogram was kept below 125 e^−^ Å^−2^ with the dose rate of ~25 e^−^ pixel^−1^ s^−1^ (Extended Data Table 1). A dose-symmetric acquisition scheme was used, in which tilt angles are recorded in an alternating pattern from 0° towards higher positive and negative tilts. At low tilt angles (including 0°), each projection was acquired with 3 movie frames, each receiving approximately 0.5 e^−^ Å^−2^, totalling ~1.5 e^−^ Å^−2^ per tilt. To compensate for the increased effective thickness and reduced contrast at higher tilt angles, we applied tilt-dependent dose weighting, based on a cosine correction model implemented in SerialEM, increasing the total dose per tilt at steeper angles proportionally to the apparent path length of the electron beam through the specimen. The dose per frame remained constant, with higher tilt angles acquired using more frames, up to five. The defocus range was between −1 μm and −5 μm.

### Cryo-ET reconstruction, segmentation and analysis

Tilt series were motion-corrected and filtered according to their cumulative dose using MOTIONCOR2^67^. The tilt series were aligned using the IMOD ETOMO package^68^. Tomograms were reconstructed without binning from aligned stacks as weighted back-projection in IMOD. The contrast of the tomograms was increased by binning the tomograms four times and applying a MATLAB-based deconvolution filter^69^. The binned and deconvoluted tomograms were manually segmented using AMIRA (ThermoFisher Scientific) or 3DMOD (IMOD). For subtomogram analysis of F-actin within stress fibres for validation, RELION 5.0^70^ was used throughout the workflow (Extended Data Fig. 6). Tilt series alignment and tomographic reconstructions were performed within RELION. A total of 1,555 segments of the filaments with 90% overlap, with a box size of 514 Å were manually selected from the area of interest using IMOD, and the corresponding coordinates were imported into RELION for subtomogram extraction in the form of a 2D stack. For subtomogram averaging, an initial cylinder reference of 40 Å diameter was used, followed by 3D classification to create an initial model with a feature. Further refinement was performed using all particles with a combination of classification and refinement schemes. The known helical parameter of F-actin was not applied as the averaging was for a validation purpose, yielding a nominal resolution of 27.7 Å. Contrast transfer function (CTF) correction was applied only during subtomographic analysis, following 3D reconstruction using RELION. Finally, the known structure of F-actin (PDB: 8A2T) was fitted to validate that the filament indeed follows the actin feature.

For Fig. 5b, the tubulin spirals were traced in the IMOD software. For Fig. 5c, intensity profiles of the individual units in microtubules, F-actin and tubulin spirals were plotted using the ‘line scan-plot profile’ option in ImageJ, and the intermolecular spacing was measured as the distance between the corresponding peaks of a given line scan. The diameter and width of the tubulin spirals was measured using ImageJ.

### Cryo-EM data collection and processing for in situ microtubule SPA

Videos of microtubules beyond axon injury sites were acquired on a Titan Krios TEM, equipped with a Gatan Bioquantum energy filter and a K3 Summit direct detector at an acceleration voltage of 300 kV. The dose rate on the camera was set to 23 e^−^ Å^−2^ s^−1^. Images were collected using SerialEM in super-resolution mode at 81,000× magnification, corresponding to a pixel size of 1.099 Å, using a defocus range from −0.8 to −2.2 μm. Images were acquired as 54-frame movies, accumulating a total electron dose of 53 e^−^ Å^−2^. A total of 4,398 holes were manually selected, resulting in the acquisition of 4,034 images (Extended Data Table 1). Images were motion-corrected with MOTIONCOR2. From those, 587 holes contained microtubules for analysis, corresponding to 396 cumulative µm of microtubules.

Microtubule 3D reconstructions were performed using CryoSPARC 4.5^71^ and FREALIGN 9.11^72^ (Extended Data Fig. 4). The 4,034 images were imported and the CTF was estimated prior to the processing. Microtubules were traced and segmented using the ‘filament tracer’ module with overlapping boxes of 82.5 Å step size, corresponding to the length of one tubulin heterodimer. A total of 399,396 boxes (640 pixels, 703 Å) were extracted and downsampled to 512 pixels (1.3738 Å per pixel), then 2× binned for faster processing. 2D classification was performed twice to discard particles with no filamentous features, resulting in 129,110 particles. The particles were classified by 3D classification with the templates of known microtubule architectures^37^, showing all analysed microtubules (1,663 tubules) have a 13-protofilament arrangement. 3D reconstruction was performed using homologous refinement, yielding a map at 5.6 Å resolution.

The alignment parameters were imported to FREALIGN for pseudohelical refinement (twist −27.64°, rise 9.695 Å) and an established seam detection protocol^73^. Particles with ambiguous seam assignments were excluded. The resulting alignment parameters were reimported back to CryoSPARC for final refinement. Final reconstructions were generated using 114,673 unbinned particles without applying microtubule-specific pseudohelical symmetry (C1 reconstruction). A combination of local refinement and per-particle defocus refinement yielded a global resolution of 3.54 Å. Focused refinement on 2 protofilaments covering 200 Å segment improved the resolution to 3.19 Å. These reconstructions showed key features including the S9–S10 loop that distinguishes α- and β-tubulin and clear EpoB density with clear topological detail. The final map without sharpening was used for display in Fig. 3e, and the conventionally B-factor sharpened map (B-factor −68.3 Å) was shown in Fig. 3f. The local resolution was estimated using CryoSPARC.

Intra-dimer and inter-dimer spacing within microtubules were measured as follows. First, a reference model consisting of two αβ-tubulin dimers (PDB: 3JAS, chains A, B, K and H, renamed to chains A–D) fitted into the cryo-EM density map using the fit-in-map tool in Chimera. The model was aligned opposite to the seam side to allow canonical lattice fitting. Next, individual chains were separated using the split command. Each tubulin monomer (α or β) was then independently refined against the density map using the fit-in-map tool. These four monomers were used for the distance calculation. To extract distances, we measured median distances between corresponding Cα atoms across tubulin subunits: intra-dimer distances were calculated between chains A (α-tubulin) and B (β-tubulin) within a single dimer. Inter-dimer distances were calculated between chains B (β-tubulin) and C (α-tubulin) of adjacent dimers. To avoid bias from flexible regions, only Cα atoms from structurally conserved regions (residues 1–37 and 48–170, excluding loop residues) were considered in the distance calculations.

### Model building and refinement

The initial model was prepared using PDB code 6DPU, fit into the cryo-EM map using ChimeraX^74^ and further refined by rounds of manual fitting in COOT^75^ with subsequent real-space refinement in PHENIX^76^. The map had clear density for EpoB, and the initial coordinates were taken from the monomer library (EPB). The protein sequence was assigned to mouse TUBA1A (Uniprot P68369) and TUBB3 (Uniprot Q9ERD7), respectively, as they are the most prominent isotypes in brain tissue and provided the best fit. The structure showed GTP bound to α-tubulin and GDP bound to β-tubulin. Isotropic B-factors were assigned in phenix.refine. The final model had a MolProbity score of 1.429 with a correlation coefficient CC(mask) of 0.83 and an FSC(model, masked) of 0.5 at 3.61 Å and of 0.143 at 3.17 Å. As a note, the peptide bonds of P274 in α-tubulin and P272 in β-tubulin showed *cis* configuration. Data collection, reconstruction and refinement statistics are given in Extended Data Table 1.

### MIP analysis

The MIP detection in tomograms was performed using an in-house workflow based on the procedure described previously^43^ and the scheme given in Extended Data Fig. 6a. The workflow is divided into three steps: microtubule tracing, density tracing inside microtubules and MIP particle picking.

Microtubules were segmented using a semi-automated approach^77^ available in the Amira 2021.2 software (Thermo Fisher Scientific) using a hollow cylinder as a template with 100 nm length, 7 nm radius for lumen, and 15 nm radius for external shell. The coordinates of the traced filaments were resampled using a cubic interpolation to provide a uniform and dense representation of the centre lines.

Densities were template-free picked in 3D by adapting the Python package PySeg^78^ for processing MIPs within microtubules. PySeg utilizes discrete Morse theory to analyse all densities in a volume independently of their size or shape and sort them by their relevance (topological persistence). Firstly, tomograms were smoothed by a Gaussian low-pass filtering at *σ*_v_ = 0.5 pixels. Secondly, a mask for the microtubule lumen was generated; the lumen volume was segmented around the previously computed microtubule centre line by the distance transform. Finally, Pyseg was configured for processing only the volume within lumen masks. Originally, PySeg was designed to trace densities and their interconnections. Here we were only interested in picking lumenal particles independently of interconnections or associations within the microtubule shell. Therefore, connectivity information was only used to sort densities by their topological persistence. Greyscale minima positions were identified and the most relevant ones (higher topological persistence) were preserved by topological persistence simplifications, while discarding those generated from noise or spurious densities as described^79^. The persistence threshold was adapted to consider the different contrast conditions for each tomogram. Since the microtubule shell is a polymer of tubulins with a regular density, it is expected that the number of minima per volume unit (density) for every microtubule in the dataset is constant. Consequently, the persistence threshold was adjusted adaptively to obtain the same density of minima at the shell for every microtubule, specifically 0.006 vertices per nm^3^. The shell volume was computed similarly to the lumen, but increasing the distance to the centre line according to the shell’s external radius and subtracting the lumen. MIPs typically have a diameter of 6 nm; we used the mean shift clustering method^80^ with a bandwidth set to 5 nm to avoid over-picking inherent to complexes composed of several visible domains (density minima). In addition, we visually supervised several microtubules to ensure that MIPs were associated with a single picked particle (Extended Data Fig. 6a).

### Microtubule polarity

The polarities of individual microtubules were obtained by the 3D alignment process in RELION during microtubule SPA. Rotations of individual microtubule segments relative to a reference density (EMD-0614) were extracted. The average rotation value was calculated among all microtubule segments in a single image and used as a reference axis. The absolute values of the rotations normalized by the reference axis were plotted to assess the distributions of polarities.

### Membrane tension analysis

Thalamus explant tissue plated on grid-etched glass-bottom dishes were used to perform axotomy as described above. Axons after the injury were incubated with 1 nM EpoB for 1 h in a cell culture incubator. The glass-bottom dish with the explants were treated with 1 µM of Flipper-TR (CY-SC020) for 5 min in the microscope’s imaging chamber set at 37 °C and 5% CO_2_. The regenerating axons on the dish were located with the help of a photoetched grid on the 35 mm glass-bottom dish. Single *z*-plane FLIM images of the regenerating axons were acquired using a Leica SP8 Falcon FLIM confocal microscope, with a 40× or 60× objective and the Leica Las X (v.3.5.7) software. Point scanning excitation was performed at 488 nm using a pulsed white-light laser with emission collected over a bandwidth of 550–650 nm onto a hybrid single molecule detector (HyD SMD) in single photon counting mode with a pinhole set to 1 A.U. A format of 512 × 512 allowed for pixel sizes of 0.144 (40×) or 0.180 (60×). The fluorescence from regenerating axons was measured as frames accumulated over 30–50 images to obtain enough photons per pixel for subsequent analysis. Initial lifetime fitting showed that a bin of two provided counts in the range of 600−1,000 photons per pixel and that decays were well fit by a dual exponential, as reported in the literature^81^. Final FLIM image quantification was performed by transforming fluorescence decay curves into phasor space using transformation algorithms in the IDL (NV5 Geospatial). Average lifetime values from regions of interest were then computed from the centres of the phasor clouds for each region of interest. ‘Regenerating front’ or ‘growth cone’ is defined as the very end of the axon, and ‘axon shaft’ was defined as a region approximately 15 µm away from the tip of the axon. The data were plotted using GraphPad Prism.

### Tubulin Tracker binding

The binding ability of the Tubulin Tracker dye to tubulin monomers, microtubules and tubulin spirals^44^ induced by p150 fragment 1–105 was determined by measuring the emission spectra of the dye when it was bound to the substrate. The p150 protein was purified using the protocol described previously^44^. Tubulin was purified using cycles of polymerization and depolymerization from porcine brains^44^. Microtubules were polymerized at 37 °C using a final concentration of 1 mM GTP in 1× BRB80 buffer (80 mM PIPES-KOH pH 6.8, 1 mM MgCl_2_, 1 mM EGTA) for 30 min. Tubulin was mixed with different concentrations (10 µM and 100 µM) of p150 as specified in the figure. 200 nM of Tubulin Tracker was added to each well of a Corning 384-well black plate. The absorbance was measured using a BMG CLATIOstar+ plate reader and plotted using GraphPad Prism.

### Statistics and reproducibility

Sample sizes were not predetermined using statistical methods. The study utilized random assignment of samples in which each sample received a unique treatment or condition. All attempts of replication, both technical and biological were successful. All experiments were performed and analysed from at least three biologically independent experiments except for Extended Data Figs. 5c and 8b,c, for which experiments were performed two times. Calculations of the raw data were performed in Microsoft Excel 15.35, MATLAB 2021b (MathWorks) and the final data were plotted using GraphPad PRISM 7. Significance tests used for each comparison are mentioned in the respective figure legends.

### Reporting summary

Further information on research design is available in the Nature Portfolio Reporting Summary linked to this article.

## Online content

Any methods, additional references, Nature Portfolio reporting summaries, source data, extended data, supplementary information, acknowledgements, peer review information; details of author contributions and competing interests; and statements of data and code availability are available at 10.1038/s41586-025-09654-z.

## Supplementary information

Reporting Summary

Supplementary Video 1 Representative live image of axotomy performed on an EM grid. Yellow arrows indicate the initial location of the target axon, which is cut and partially removed. Scale bar: 50 µm.

Supplementary Video 2 Axon response after axotomy. Axon growth after axotomy (marked with yellow arrowheads, left). Stalled or retracting axons after axotomy (marked with green arrowheads, right). Scale bar: 20 µm.

Supplementary Video 3 Regeneration of thalamus axons. Regeneration of thalamus axons after axotomy is tested under different doses of EpoB treatment. Scale bar: 10 µm.

Supplementary Video 4 Taxol induced regeneration. Scale bar: 20 µm.

Supplementary Video 5 Regeneration of thalamus axons after axotomy in presence of 1 nM EpoB. Scale bar: 5 µm. Cyan – Microtubules, Actin – Magenta, Plasma Membrane – Yellow.

Supplementary Video 6 Cryo-ET reconstruction of microtubules in the regenerating axon 1 h after axotomy in the presence of 1 nM EpoB. Scale bar: 100 nm.

Supplementary Video 7 EB3-labelled microtubule plus-end growth in regenerating thalamus axons. EB3-labelled (in red) microtubule growing plus ends in thalamus neurons with or without axotomy treated with EpoB and recorded at different times were shown in the movies. Axotomy sites are shown as a dotted white line. Scale bar: 10 µm.

Supplementary Video 8 Tubulin movement in thalamus neurons with or without axotomy and EpoB. Tubulin Tracker^TM^-labelled tubulin movements in thalamus neurons with or without axotomy treated with or without EpoB were recorded for a period of 30 minutes. Axotomy sites are shown as a dotted white line. Scale bar: 10 µm.

Supplementary Video 9 Tubulin movements in thalamus neurons treated with EpoB or Monastrol. Tubulin Tracker^TM^-labelled tubulin movements in thalamus neurons treated with EpoB and/or Monastrol were recorded for 10 minutes. Scale bar: 10 µm.

Supplementary Video 10 Co-localization of KIF5 with tubulin puncta. Co-localization of KIF5 motor proteins with the tubulin tracker-labelled puncta was observed. Tubulin Tracker and Kinesin signals were shown in Cyan and Magenta, respectively. Arrow heads show the co-localization examples. Scale bar - 5 µm.

Supplementary Video 11 Cryo-ET reconstruction of a regenerating thalamus axon in the presence of 1 nM EpoB showing a cluster of tubulin-oligomers. Scale bar: 100 nm.

Supplementary Video 12 Cryo-ET reconstruction of a regenerating thalamus axon in the presence of 1 nM EpoB, showing a microtubule branch. Snapshots of sequential slices are shown to highlight branching microtubules. Scale bar: 200 nm.

## Source data

Source Data Fig. 1, 2, 3, 4, 5 and Source Data Extended Data Fig. 1, 5, 7, 8

## Figures and Tables

**Extended Data Table 1:**
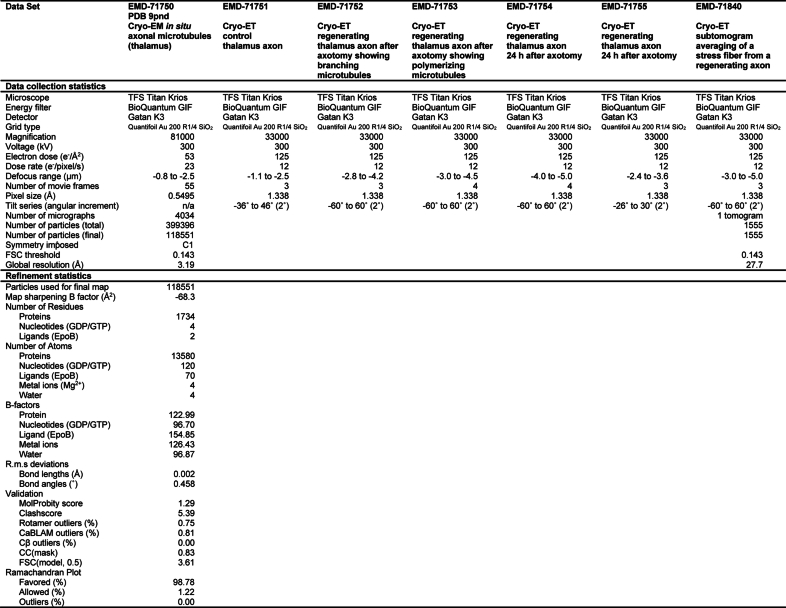
Data collection, reconstruction, and refinement statistics

## Data Availability

Tomograms used in the figures were deposited to the Electron Microscopy Database (EMDB) with accession codes EMD-71751 (regular axon), EMD-71752 (regenerating axon after axotomy showing branching microtubules), EMD-71753 (regenerating axon after axotomy showing polymerizing microtubules), EMD-71754 and EMD-71755 (regenerating axons after axotomy). The cryo-EM map from subtomogram averaging of actin stress fibres is available as EMD-71840. The SPA reconstruction of in situ EpoB-induced microtubules is available under accession code EMD-71750, and the coordinates of the final model under PDB code 9PND. Source data are provided with this paper.

## References

[1] Curcio, M. & Bradke, F. Axon regeneration in the central nervous system: facing the challenges from the inside. *Annu. Rev. Cell Dev. Biol.* **34**, 495–521 (2018). 30044649 10.1146/annurev-cellbio-100617-062508

[2] He, Z. & Jin, Y. Intrinsic control of axon regeneration. *Neuron* **90**, 437–451 (2016). 27151637 10.1016/j.neuron.2016.04.022

[3] Fawcett, J. W. The struggle to make CNS axons regenerate: why has it been so difficult? *Neurochem. Res.* **45**, 144–158 (2020). 31388931 10.1007/s11064-019-02844-y PMC6942574

[4] Sharp, D. J., Scott, G. & Leech, R. Network dysfunction after traumatic brain injury. *Nat. Rev. Neurol.* **10**, 156–166 (2014). 24514870 10.1038/nrneurol.2014.15

[5] Bradke, F., Fawcett, J. W. & Spira, M. E. Assembly of a new growth cone after axotomy: the precursor to axon regeneration. *Nat. Rev. Neurosci.* **13**, 183–193 (2012). 22334213 10.1038/nrn3176

[6] Schlaepfer, W. W. Calcium-induced degeneration of axoplasm in isolated segments of rat peripheral nerve. *Brain Res.* **69**, 203–215 (1974). 4823092 10.1016/0006-8993(74)90002-x

[7] Wolf, J. A., Stys, P. K., Lusardi, T., Meaney, D. & Smith, D. H. Traumatic axonal injury induces calcium influx modulated by tetrodotoxin-sensitive sodium channels. *J. Neurosci.* **21**, 1923–1930 (2001). 11245677 10.1523/JNEUROSCI.21-06-01923.2001 PMC6762603

[8] Ziv, N. E. & Spira, M. E. Axotomy induces a transient and localized elevation of the free intracellular calcium concentration to the millimolar range. *J. Neurophysiol.* **74**, 2625–2637 (1995). 8747220 10.1152/jn.1995.74.6.2625

[9] Schlaepfer, W. W. & Bunge, R. P. Effects of calcium ion concentration on the degeneration of amputated axons in tissue culture. *J. Cell Biol.* **59**, 456–470 (1973). 4805010 10.1083/jcb.59.2.456 PMC2109098

[10] Ramon y Cajal, S. & May, R. M. *Degeneration and Regeneration of the Nervous System* (Oxford Univ. Press, 1928).

[11] Li, D., Field, P. M. & Raisman, G. Failure of axon regeneration in postnatal rat entorhinohippocampal slice coculture is due to maturation of the axon, not that of the pathway or target. *Eur. J. Neurosci.* **7**, 1164–1171 (1995). 7582089 10.1111/j.1460-9568.1995.tb01106.x

[12] Tang-Schomer, M. D., Patel, A. R., Baas, P. W. & Smith, D. H. Mechanical breaking of microtubules in axons during dynamic stretch injury underlies delayed elasticity, microtubule disassembly, and axon degeneration. *FASEB J.* **24**, 1401–1410 (2010). 20019243 10.1096/fj.09-142844 PMC2879950

[13] Erturk, A., Hellal, F., Enes, J. & Bradke, F. Disorganized microtubules underlie the formation of retraction bulbs and the failure of axonal regeneration. *J. Neurosci.* **27**, 9169–9180 (2007). 17715353 10.1523/JNEUROSCI.0612-07.2007 PMC6672197

[14] Blanquie, O. & Bradke, F. Cytoskeleton dynamics in axon regeneration. *Curr. Opin. Neurobiol.* **51**, 60–69 (2018). 29544200 10.1016/j.conb.2018.02.024

[15] Tedeschi, A. et al. ADF/cofilin-mediated actin turnover promotes axon regeneration in the adult CNS. *Neuron* **103**, 1073–1085.e1076 (2019). 31400829 10.1016/j.neuron.2019.07.007 PMC6763392

[16] Stern, S. et al. RhoA drives actin compaction to restrict axon regeneration and astrocyte reactivity after CNS injury. *Neuron* **109**, 3436–3455.e9 (2021). 34508667 10.1016/j.neuron.2021.08.014

[17] Wu, D. et al. Chronic neuronal activation increases dynamic microtubules to enhance functional axon regeneration after dorsal root crush injury. *Nat. Commun.* **11**, 6131 (2020). 33257677 10.1038/s41467-020-19914-3 PMC7705672

[18] Hellal, F. et al. Microtubule stabilization reduces scarring and causes axon regeneration after spinal cord injury. *Science* **331**, 928–931 (2011). 21273450 10.1126/science.1201148 PMC3330754

[19] Sengottuvel, V., Leibinger, M., Pfreimer, M., Andreadaki, A. & Fischer, D. Taxol facilitates axon regeneration in the mature CNS. *J. Neurosci.* **31**, 2688–2699 (2011). 21325537 10.1523/JNEUROSCI.4885-10.2011 PMC6623712

[20] Ruschel, J. et al. Systemic administration of epothilone B promotes axon regeneration after spinal cord injury. *Science* **348**, 347–352 (2015). 25765066 10.1126/science.aaa2958 PMC4445125

[21] Brunden, K. R. et al. The characterization of microtubule-stabilizing drugs as possible therapeutic agents for Alzheimer’s disease and related tauopathies. *Pharmacol. Res.* **63**, 341–351 (2011). 21163349 10.1016/j.phrs.2010.12.002 PMC3042036

[22] Nettles, J. H. et al. The binding mode of epothilone A on alpha,beta-tubulin by electron crystallography. *Science* **305**, 866–869 (2004). 15297674 10.1126/science.1099190

[23] Prota, A. E. et al. Molecular mechanism of action of microtubule-stabilizing anticancer agents. *Science* **339**, 587–590 (2013). 23287720 10.1126/science.1230582

[24] Howes, S. C. et al. Structural differences between yeast and mammalian microtubules revealed by cryo-EM. *J. Cell Biol.* **216**, 2669–2677 (2017). 28652389 10.1083/jcb.201612195 PMC5584162

[25] Perez, E. A. et al. Efficacy and safety of ixabepilone (BMS-247550) in a phase II study of patients with advanced breast cancer resistant to an anthracycline, a taxane, and capecitabine. *J. Clin. Oncol.* **25**, 3407–3414 (2007). 17606974 10.1200/JCO.2006.09.3849

[26] Colom, A. et al. A fluorescent membrane tension probe. *Nat. Chem.* **10**, 1118–1125 (2018). 30150727 10.1038/s41557-018-0127-3 PMC6197433

[27] Skaliora, I., Adams, R. & Blakemore, C. Morphology and growth patterns of developing thalamocortical axons. *J. Neurosci.* **20**, 3650–3662 (2000). 10804207 10.1523/JNEUROSCI.20-10-03650.2000 PMC6772677

[28] Goodson, H. V. & Jonasson, E. M. Microtubules and microtubule-associated proteins. *Cold Spring Harb. Perspect. Biol.* **10**, a022608 (2018). 29858272 10.1101/cshperspect.a022608 PMC5983186

[29] Chretien, D., Metoz, F., Verde, F., Karsenti, E. & Wade, R. H. Lattice defects in microtubules: protofilament numbers vary within individual microtubules. *J. Cell Biol.* **117**, 1031–1040 (1992). 1577866 10.1083/jcb.117.5.1031 PMC2289483

[30] Mizuno, N. et al. Dynein and kinesin share an overlapping microtubule-binding site. *EMBO J.* **23**, 2459–2467 (2004). 15175652 10.1038/sj.emboj.7600240 PMC449763

[31] Baas, P. W., Rao, A. N., Matamoros, A. J. & Leo, L. Stability properties of neuronal microtubules. *Cytoskeleton* **73**, 442–460 (2016). 26887570 10.1002/cm.21286 PMC5541393

[32] Moores, C. A. et al. Mechanism of microtubule stabilization by doublecortin. *Mol. Cell* **14**, 833–839 (2004). 15200960 10.1016/j.molcel.2004.06.009

[33] Tymanskyj, S. R. & Ma, L. MAP7 prevents axonal branch retraction by creating a stable microtubule boundary to rescue polymerization. *J. Neurosci.* **39**, 7118–7131 (2019). 31391261 10.1523/JNEUROSCI.0775-19.2019 PMC6733548

[34] Heidemann, S. R., Landers, J. M. & Hamborg, M. A. Polarity orientation of axonal microtubules. *J. Cell Biol.* **91**, 661–665 (1981). 6173385 10.1083/jcb.91.3.661 PMC2112798

[35] Burton, P. R. & Paige, J. L. Polarity of axoplasmic microtubules in the olfactory nerve of the frog. *Proc. Natl Acad. Sci. USA* **78**, 3269–3273 (1981). 6973153 10.1073/pnas.78.5.3269 PMC319543

[36] Baas, P. W. & Lin, S. Hooks and comets: the story of microtubule polarity orientation in the neuron. *Dev. Neurobiol.* **71**, 403–418 (2011). 21557497 10.1002/dneu.20818 PMC3151545

[37] Zhang, R., LaFrance, B. & Nogales, E. Separating the effects of nucleotide and EB binding on microtubule structure. *Proc. Natl Acad. Sci. USA* **115**, E6191–E6200 (2018). 29915050 10.1073/pnas.1802637115 PMC6142192

[38] Itzhak, D. N., Tyanova, S., Cox, J. & Borner, G. H. Global, quantitative and dynamic mapping of protein subcellular localization. *eLife* **5**, e16950 (2016). 27278775 10.7554/eLife.16950 PMC4959882

[39] Hiller, G. & Weber, K. Radioimmunoassay for tubulin: a quantitative comparison of the tubulin content of different established tissue culture cells and tissues. *Cell* **14**, 795–804 (1978). 688394 10.1016/0092-8674(78)90335-5

[40] Nedozralova, H. et al. In situ cryo-electron tomography reveals local cellular machineries for axon branch development. *J. Cell Biol.* **221**, e202106086 (2022). 35262630 10.1083/jcb.202106086 PMC8916118

[41] Myers, K. A. & Baas, P. W. Kinesin-5 regulates the growth of the axon by acting as a brake on its microtubule array. *J. Cell Biol.* **178**, 1081–1091 (2007). 17846176 10.1083/jcb.200702074 PMC2064629

[42] Cuveillier, C. et al. MAP6 is an intraluminal protein that induces neuronal microtubules to coil. *Sci. Adv.* **6**, eaaz4344 (2020). 32270043 10.1126/sciadv.aaz4344 PMC7112752

[43] Chakraborty, S. et al. Cryo-ET suggests tubulin chaperones form a subset of microtubule lumenal particles with a role in maintaining neuronal microtubules. *Proc. Natl Acad. Sci. USA* **122**, e2404017121 (2025). 39888918 10.1073/pnas.2404017121 PMC11804619

[44] Wang, Q., Crevenna, A. H., Kunze, I. & Mizuno, N. Structural basis for the extended CAP-Gly domains of p150(glued) binding to microtubules and the implication for tubulin dynamics. *Proc. Natl Acad. Sci. USA* **111**, 11347–11352 (2014). 25059720 10.1073/pnas.1403135111 PMC4128117

[45] Ayukawa, R. et al. GTP-dependent formation of straight tubulin oligomers leads to microtubule nucleation. *J. Cell Biol.* **220**, e202007033 (2021). 33544140 10.1083/jcb.202007033 PMC7871348

[46] McIntosh, J. R. et al. Microtubules grow by the addition of bent guanosine triphosphate tubulin to the tips of curved protofilaments. *J. Cell Biol.* **217**, 2691–2708 (2018). 29794031 10.1083/jcb.201802138 PMC6080942

[47] Mandelkow, E. M., Mandelkow, E. & Milligan, R. A. Microtubule dynamics and microtubule caps: a time-resolved cryo-electron microscopy study. *J. Cell Biol.* **114**, 977–991 (1991). 1874792 10.1083/jcb.114.5.977 PMC2289108

[48] Ojeda-Lopez, M. A. et al. Transformation of Taxol-stabilized microtubules into inverted tubulin tubules triggered by a tubulin conformation switch. *Nat. Mater.* **13**, 195–203 (2014). 24441880 10.1038/nmat3858 PMC3946914

[49] Basnet, N. et al. Direct induction of microtubule branching by microtubule nucleation factor SSNA1. *Nat. Cell Biol.* **20**, 1172–1180 (2018). 30250060 10.1038/s41556-018-0199-8 PMC6330057

[50] Zhang, B. et al. Synaptic vesicle size and number are regulated by a clathrin adaptor protein required for endocytosis. *Neuron* **21**, 1465–1475 (1998). 9883738 10.1016/s0896-6273(00)80664-9

[51] Silver, J., Schwab, M. E. & Popovich, P. G. Central nervous system regenerative failure: role of oligodendrocytes, astrocytes, and microglia. *Cold Spring Harb. Perspect. Biol.* **7**, a020602 (2014). 25475091 10.1101/cshperspect.a020602 PMC4355267

[52] Richardson, P. M., McGuinness, U. M. & Aguayo, A. J. Axons from CNS neurons regenerate into PNS grafts. *Nature* **284**, 264–265 (1980). 7360259 10.1038/284264a0

[53] Liu, K., Tedeschi, A., Park, K. K. & He, Z. Neuronal intrinsic mechanisms of axon regeneration. *Annu. Rev. Neurosci.* **34**, 131–152 (2011). 21438684 10.1146/annurev-neuro-061010-113723

[54] Cho, Y., Sloutsky, R., Naegle, K. M. & Cavalli, V. Injury-induced HDAC5 nuclear export is essential for axon regeneration. *Cell* **155**, 894–908 (2013). 24209626 10.1016/j.cell.2013.10.004 PMC3987749

[55] Rishal, I. & Fainzilber, M. Axon-soma communication in neuronal injury. *Nat. Rev. Neurosci.* **15**, 32–42 (2014). 24326686 10.1038/nrn3609

[56] Varadarajan, S. G., Hunyara, J. L., Hamilton, N. R., Kolodkin, A. L. & Huberman, A. D. Central nervous system regeneration. *Cell* **185**, 77–94 (2022). 34995518 10.1016/j.cell.2021.10.029 PMC10896592

[57] Tian, F. et al. Core transcription programs controlling injury-induced neurodegeneration of retinal ganglion cells. *Neuron* **110**, 2607–2624.e2608 (2022). 35767995 10.1016/j.neuron.2022.06.003 PMC9391318

[58] Moeendarbary, E. et al. The soft mechanical signature of glial scars in the central nervous system. *Nat. Commun.* **8**, 14787 (2017). 28317912 10.1038/ncomms14787 PMC5364386

[59] Giger, R. J., Hollis, E. R. 2nd & Tuszynski, M. H. Guidance molecules in axon regeneration. *Cold Spring Harb. Perspect. Biol.* **2**, a001867 (2010). 20519341 10.1101/cshperspect.a001867 PMC2890195

[60] Gallo, V. & Deneen, B. Glial development: the crossroads of regeneration and repair in the CNS. *Neuron* **83**, 283–308 (2014). 25033178 10.1016/j.neuron.2014.06.010 PMC4114724

[61] Farias, G. G., Guardia, C. M., Britt, D. J., Guo, X. & Bonifacino, J. S. Sorting of dendritic and axonal vesicles at the pre-axonal exclusion zone. *Cell Rep.* **13**, 1221–1232 (2015). 26527003 10.1016/j.celrep.2015.09.074 PMC5410646

[62] Yang, R. et al. A novel strategy to visualize vesicle-bound kinesins reveals the diversity of kinesin-mediated transport. *Traffic* **20**, 851–866 (2019). 31461551 10.1111/tra.12692 PMC7714429

[63] Dunn, S. et al. Differential trafficking of Kif5c on tyrosinated and detyrosinated microtubules in live cells. *J. Cell Sci.* **121**, 1085–1095 (2008). 18334549 10.1242/jcs.026492

[64] Schindelin, J. et al. Fiji: an open-source platform for biological-image analysis. *Nat. Methods* **9**, 676–682 (2012). 22743772 10.1038/nmeth.2019 PMC3855844

[65] Bodakuntla, S., Magiera, M. M. & Janke, C. Measuring the impact of tubulin posttranslational modifications on axonal transport. *Methods Mol. Biol.* **2101**, 353–370 (2020). 31879913 10.1007/978-1-0716-0219-5_20

[66] Hagen, W. J. H., Wan, W. & Briggs, J. A. G. Implementation of a cryo-electron tomography tilt-scheme optimized for high resolution subtomogram averaging. *J. Struct. Biol.* **197**, 191–198 (2017). 27313000 10.1016/j.jsb.2016.06.007 PMC5287356

[67] Zheng, S. Q. et al. MotionCor2: anisotropic correction of beam-induced motion for improved cryo-electron microscopy. *Nat. Methods* **14**, 331–332 (2017). 28250466 10.1038/nmeth.4193 PMC5494038

[68] Mastronarde, D. N. & Held, S. R. Automated tilt series alignment and tomographic reconstruction in IMOD. *J. Struct. Biol.* **197**, 102–113 (2017). 27444392 10.1016/j.jsb.2016.07.011 PMC5247408

[69] Tegunov, D. & Cramer, P. Real-time cryo-electron microscopy data preprocessing with Warp. *Nat. Methods* **16**, 1146–1152 (2019). 31591575 10.1038/s41592-019-0580-y PMC6858868

[70] Scheres, S. H. RELION: implementation of a Bayesian approach to cryo-EM structure determination. *J. Struct. Biol.* **180**, 519–530 (2012). 23000701 10.1016/j.jsb.2012.09.006 PMC3690530

[71] Punjani, A., Rubinstein, J. L., Fleet, D. J. & Brubaker, M. A. cryoSPARC: algorithms for rapid unsupervised cryo-EM structure determination. *Nat. Methods* **14**, 290–296 (2017). 28165473 10.1038/nmeth.4169

[72] Grigorieff, N. Frealign: an exploratory tool for single-particle Cryo-EM. *Methods Enzymol.* **579**, 191–226 (2016). 27572728 10.1016/bs.mie.2016.04.013 PMC6760665

[73] Zhang, R. & Nogales, E. A new protocol to accurately determine microtubule lattice seam location. *J. Struct. Biol.* **192**, 245–254 (2015). 26424086 10.1016/j.jsb.2015.09.015 PMC4634897

[74] Meng, E. C. et al. UCSF ChimeraX: tools for structure building and analysis. *Protein Sci.* **32**, e4792 (2023). 37774136 10.1002/pro.4792 PMC10588335

[75] Emsley, P., Lohkamp, B., Scott, W. G. & Cowtan, K. Features and development of Coot. *Acta Crystallogr. D* **66**, 486–501 (2010). 20383002 10.1107/S0907444910007493 PMC2852313

[76] Afonine, P. V. et al. Real-space refinement in PHENIX for cryo-EM and crystallography. *Acta Crystallogr. D* **74**, 531–544 (2018). 10.1107/S2059798318006551 PMC6096492 29872004

[77] Rusu, M., Starosolski, Z., Wahle, M., Rigort, A. & Wriggers, W. Automated tracing of filaments in 3D electron tomography reconstructions using Sculptor and Situs. *J. Struct. Biol.* **178**, 121–128 (2012). 22433493 10.1016/j.jsb.2012.03.001 PMC3440181

[78] Martinez-Sanchez, A. et al. Template-free detection and classification of membrane-bound complexes in cryo-electron tomograms. *Nat. Methods* **17**, 209–216 (2020). 31907446 10.1038/s41592-019-0675-5

[79] Sousbie, T. The persistent cosmic web and its filamentary structure - I. Theory and implementation. *Mon. Not. R. Astron. Soc.* **414**, 350–383 (2011).

[80] Comaniciu, D. & Meer, P. Mean shift: a robust approach toward feature space analysis. *IEEE Trans. Pattern Anal. Mach. Intell.* **24**, 603–619 (2002).

[81] Digman, M. A., Caiolfa, V. R., Zamai, M. & Gratton, E. The phasor approach to fluorescence lifetime imaging analysis. *Biophys. J.* **94**, L14–L16 (2008). 17981902 10.1529/biophysj.107.120154 PMC2157251

