# Supplementary material for: In situ structural mechanism of epothilone-B-induced CNS axon regeneration
Source: Nature. 2025 Nov 12;648(8093):477–87. doi: 10.1038/s41586-025-09654-z (PMC12795760; doi:10.1038/s41586-025-09654-z)
Supplement: Supplementary file 1 — Reporting Summary [file 41586_2025_9654_MOESM1_ESM.pdf]

nature portfolio

Corresponding author(s): Naoko Mizuno

Last updated by author(s): Sep 11, 2025

Reporting Summary

Nature Portfolio wishes to improve the reproducibility of the work that we publish. This form provides structure for consistency and transparency in reporting. For further information on Nature Portfolio policies, see our Editorial Policies and the Editorial Policy Checklist.

Statistics

For all statistical analyses, confirm that the following items are present in the figure legend, table legend, main text, or Methods section.

n/a	Confirmed	
☐	☒	The exact sample size (*n*) for each experimental group/condition, given as a discrete number and unit of measurement
☐	☒	A statement on whether measurements were taken from distinct samples or whether the same sample was measured repeatedly
☐	☒	The statistical test(s) used AND whether they are one- or two-sided<br>*Only common tests should be described solely by name; describe more complex techniques in the Methods section.*
☐	☒	A description of all covariates tested
☐	☒	A description of any assumptions or corrections, such as tests of normality and adjustment for multiple comparisons
☐	☒	A full description of the statistical parameters including central tendency (e.g. means) or other basic estimates (e.g. regression coefficient) AND variation (e.g. standard deviation) or associated estimates of uncertainty (e.g. confidence intervals)
☐	☒	For null hypothesis testing, the test statistic (e.g. *F*, *t*, *r*) with confidence intervals, effect sizes, degrees of freedom and *P* value noted<br>*Give P values as exact values whenever suitable.*
☒	☐	For Bayesian analysis, information on the choice of priors and Markov chain Monte Carlo settings
☒	☐	For hierarchical and complex designs, identification of the appropriate level for tests and full reporting of outcomes
☒	☐	Estimates of effect sizes (e.g. Cohen's *d*, Pearson's *r*), indicating how they were calculated

Our web collection on statistics for biologists contains articles on many of the points above.

Software and code

Policy information about availability of computer code

Data collection	For EM-data collection of SPA and tomograms, Serial-EM 4.2 application (https://bio3d.colorado.edu/SerialEM/) was used. For immunofluorescence and live imaging, either Zeiss 2.3, or NIS elements 6 softwares were used.
Data analysis	Analysis was performed using Fiji 1.54 (https://imagej.net/software/fiji/downloads) with plugins described in the methods section.<br><br>For electron microscopy – MATLAB 2021b, RELION 5 (https://www2.mrc-lmb.cam.ac.uk/relion/), Frealign 9 .11(grigoriefflab.janelia.org/frealign), customized pipeline for microtubule analysis (PMID: 26424086), CryoSPARC 4.5 (https://cryosparc.com/) and for tomographic reconstruction IMOD 4.12 (bio3d.colorado.edu/imod/) was used.<br><br>For segmentation, AMIRA 2021.2 (ThermoFisher Scientific) and IMOD 4.12 were used.<br>For MIPs segmentation, Pyseg 2.0 (https://github.com/anmartinezs/pyseg_system) was used.<br><br>Prism 7 and Microsoft Excel 15.35 were used for statistical analysis and graphical data presentation (https://www.graphpad.com/).

For manuscripts utilizing custom algorithms or software that are central to the research but not yet described in published literature, software must be made available to editors and reviewers. We strongly encourage code deposition in a community repository (e.g. GitHub). See the Nature Portfolio guidelines for submitting code & software for further information.

Data

Policy information about availability of data

All manuscripts must include a data availability statement. This statement should provide the following information, where applicable:

- Accession codes, unique identifiers, or web links for publicly available datasets
- A description of any restrictions on data availability
- For clinical datasets or third party data, please ensure that the statement adheres to our policy

GMPCPP-stabilized microtubules (PDB: 6dpu, https://www.rcsb.org/structure/6DPU) and GDP-bound microtubules (PDB: 6dpv, https://www.rcsb.org/structure/6DPV) were used to compare with EpoB-bound microtubules.
F-actin (PDB: 8a2t, https://www.rcsb.org/structure/8A2T) was used to validate the filament subtomogram averaging.
Tubulin dimer (PDB: 3JAS, https://www.rcsb.org/structure/3JAS) was used to measure inter-dimer distance of tubulin within EpoB-bound microtubules.

Tomograms used in the figures were deposited to the Electron Microscopy Database (EMDB) with accession codes EMD-71751 (regular axon), EMD-71752 (regenerating axon after axotomy showing branching microtubules), EMD-71753 (regenerating axon after axotomy showing polymerizing microtubules), EMD-71754 and EMD-71755 (regenerating axons after axotomy). The cryo-EM map from subtomogram averaging of actin stress fibers is available as EMD-71840. The SPA reconstruction of in situ EpoB-induced microtubule is available under accession code EMD-71750, and the coordinates of the final model under PDB code 9pnd. Source data are provided with this paper.

Research involving human participants, their data, or biological material

Policy information about studies with human participants or human data. See also policy information about sex, gender (identity/presentation), and sexual orientation and race, ethnicity and racism.

Reporting on sex and gender	N/A - Human participants, their data, or biological material was not used.
Reporting on race, ethnicity, or other socially relevant groupings	N/A - Human participants, their data, or biological material was not used.
Population characteristics	N/A - Human participants, their data, or biological material was not used.
Recruitment	N/A - Human participants, their data, or biological material was not used.
Ethics oversight	N/A - Human participants, their data, or biological material was not used.

Note that full information on the approval of the study protocol must also be provided in the manuscript.

Field-specific reporting

Please select the one below that is the best fit for your research. If you are not sure, read the appropriate sections before making your selection.

☒ Life sciences ☐ Behavioural & social sciences ☐ Ecological, evolutionary & environmental sciences

For a reference copy of the document with all sections, see nature.com/documents/nr-reporting-summary-flat.pdf

Life sciences study design

All studies must disclose on these points even when the disclosure is negative.

Sample size	No formal statistical methods were used to predetermine sample size. Instead, sample sizes were chosen based on established protocols in the field. All experiments were performed in at least three independent biological replicates unless otherwise stated. For each replicate, multiple axons were analyzed, and the results were consistent across replicates. We did not make assumptions beyond the empirical reproducibility of the data. We consider these sample sizes sufficient, as the observed effects were robust and reproducible across independent experiments.
Data exclusions	None
Replication	All key findings were reproducible across both technical and biological replicates. While some individual experiments occasionally failed due to technical issues (variability in tissue preparation, culture quality, or imaging conditions), none of these failures altered the overall conclusions.
Randomization	The study employed random assignment of samples where each sample received a unique treatment or condition.
Blinding	Double-blinded analysis was not feasible due to the visibly distinct phenotypes of the experimental groups, which made blinding not possible.

Reporting for specific materials, systems and methods

We require information from authors about some types of materials, experimental systems and methods used in many studies. Here, indicate whether each material, system or method listed is relevant to your study. If you are not sure if a list item applies to your research, read the appropriate section before selecting a response.

Materials & experimental systems

n/a	Involved in the study	
☐	☒	Antibodies
☐	☒	Eukaryotic cell lines
☒	☐	Palaeontology and archaeology
☐	☒	Animals and other organisms
☒	☐	Clinical data
☒	☐	Dual use research of concern
☒	☐	Plants

Methods

n/a	Involved in the study	
☒	☐	ChIP-seq
☒	☐	Flow cytometry
☒	☐	MRI-based neuroimaging

Antibodies

Antibodies used

The information about dilution and manufacturer are mentioned in the Methods section.

Validation

Antibodies and their validation from the manufacturer's site.

Anti MAP2, 'Detect Microtubule-Associated Protein 2 (MAP2) using this Anti-Microtubule-Associated Protein 2 (MAP2) Antibody validated for use in ELISA, IC, IH, IH(P) & WB with more than 55 product citations.'

Anti-Tau1, 'Anti-Tau-1 Antibody, clone PC1C6 is an antibody against Tau-1 for use in IH & WB with more than 65 product citations.'

12G10, 'Recommended Applications: FFPE, Immunofluorescence, Immunohistochemistry, Western Blot.'

Eukaryotic cell lines

Policy information about cell lines and Sex and Gender in Research

Cell line source(s)

Lenti-X-293T (TaKaRa #632180) cells were obtained from Takara. Lenti-X-293T is a HEK293T derivative optimized for lentivirus production.

Authentication

No further authentication

Mycoplasma contamination

Cells are not mycoplasma contaminated.

Commonly misidentified lines (See ICLAC register)

Cells are not listed in the database of commonly misidentified cell lines.

Animals and other research organisms

Policy information about studies involving animals; ARRIVE guidelines recommended for reporting animal research, and Sex and Gender in Research

Laboratory animals

Cultures were performed from embryos (at E15.5) of CD-1 mice (Charles River Laboratories). CD-1 pregnant mice from CRL laboratories was obtained on E15.5, housed in the animal house for 1 -2 hours and then used for the experiments.

Wild animals

No wild animals were used

Reporting on sex

Cultures were performed from embryos (at E15.5) of CD-1 mice (Charles River Laboratories).

Field-collected samples

None

Ethics oversight

All animal procedures were approved by the Animal Care and Use Committee (ACUC) of the National Heart, Lung, and Blood Institute (NHLBI) animal protocol H-0331 in accordance with NIH research guidelines for the care and use of laboratory animals.

Note that full information on the approval of the study protocol must also be provided in the manuscript.

Plants

Seed stocks	Not applicable
Novel plant genotypes	Not applicable
Authentication	Not applicable
